# Prognostic and Functional Significant of Heat Shock Proteins (HSPs) in Breast Cancer Unveiled by Multi-Omics Approaches

**DOI:** 10.3390/biology10030247

**Published:** 2021-03-22

**Authors:** Miriam Buttacavoli, Gianluca Di Cara, Cesare D’Amico, Fabiana Geraci, Ida Pucci-Minafra, Salvatore Feo, Patrizia Cancemi

**Affiliations:** 1 Department of Biological Chemical and Pharmaceutical Sciences and Technologies (STEBICEF), University of Palermo, 90128 Palermo, Italy; miriam.buttacavoli@unipa.it (M.B.); lucadicar@gmail.com (G.D.C.); cesare.damico@unipa.it (C.D.); fabiana.geraci@unipa.it (F.G.); salvatore.feo@unipa.it (S.F.); 2 Experimental Center of Onco Biology (COBS), 90145 Palermo, Italy; pucci.ida@gmail.com

**Keywords:** breast cancer, HSPs, expression, prognosis, data mining, proteomics, miRNAs

## Abstract

**Simple Summary:**

In this study, we investigated the expression pattern and prognostic significance of the heat shock proteins (HSPs) family members in breast cancer (BC) by using several bioinformatics tools and proteomics investigations. Our results demonstrated that, collectively, HSPs were deregulated in BC, acting as both oncogene and onco-suppressor genes. In particular, two different HSP-clusters were significantly associated with a poor or good prognosis. Interestingly, the HSPs deregulation impacted gene expression and miRNAs regulation that, in turn, affected important biological pathways involved in cell cycle, DNA replication, and receptors-mediated signaling. Finally, the proteomic identification of several HSPs members and isoforms revealed much more complexity of HSPs roles in BC and showed that their expression is quite variable among patients. In conclusion, we elaborated two panels of HSPs that could be further explored as potential biomarkers for BC progression and prognosis.

**Abstract:**

Heat shock proteins (HSPs) are a well-characterized molecular chaperones protein family, classified into six major families, according to their molecular size. A wide range of tumors have been shown to express atypical levels of one or more HSPs, suggesting that they could be used as biomarkers. However, the collective role and the possible coordination of HSP members, as well as the prognostic significance and the functional implications of their deregulated expression in breast cancer (BC) are poorly investigated. Here, we used a systematic multi-omics approach to assess the HSPs expression, the prognostic value, and the underlying mechanisms of tumorigenesis in BC. By using data mining, we showed that several HSPs were deregulated in BC and significantly correlated with a poor or good prognosis. Functional network analysis of HSPs co-expressed genes and miRNAs highlighted their regulatory effects on several biological pathways involved in cancer progression. In particular, these pathways concerned cell cycle and DNA replication for the HSPs co-expressed genes, and miRNAs up-regulated in poor prognosis and Epithelial to Mesenchymal Transition (ETM), as well as receptors-mediated signaling for the HSPs co-expressed genes up-regulated in good prognosis. Furthermore, the proteomic expression of HSPs in a large sample-set of breast cancer tissues revealed much more complexity in their roles in BC and showed that their expression is quite variable among patients and confined into different cellular compartments. In conclusion, integrative analysis of multi-omics data revealed the distinct impact of several HSPs members in BC progression and indicate that collectively they could be useful as biomarkers and therapeutic targets for BC management.

## 1. Introduction

Breast cancer (BC) represents the most common type and the leading cause of death of cancer among females and accounts for ~16% of all cancers [1]. The etiology of BC is quite complex, involving several genetic and epigenetic changes [2]. Consequently, breast cancer is a heterogeneous disease with several subtypes of different cellular compositions, molecular alterations, as well as clinical behavior [3,4]. Several factors, such as histological grade, type and size of the tumor, lymph node metastasis, estrogen receptor (ER), progesterone receptor (PR), and human epidermal growth factor receptor 2 (HER), are generally considered as prognostic factors, but are insufficient to provide useful information for clinical management [5,6]. Despite the achieved improvements, the prognosis of BC patients is still a poor predictor and the identification of more reliable biomarkers must be explored.

Heat shock proteins (HSPs) are one of the largest groups of molecular chaperones that assist the correct folding of partially folded or denatured proteins and prevent the formation of potential aggregates in the cells, promoting their proteasomal degradation [7]. HSPs were first discovered as stress-inducible proteins against physical (temperature elevation) or chemical (increase or decrease in pH, salinity, or oxygen concentration) stressors, performing a wide range of functional activities: Modulation of their synthesis, regulation of kinases activation, participation in signal transduction pathways and rRNA processing [8]. Under physiological conditions, HSPs perform chaperone functions on a broad array of client proteins, including transcription factors, nuclear hormone receptors, viral proteins and signaling mediators. Depending on their molecular weight and main functions, six major subfamilies of HSPs have been reported: HSP110, HSP90, HSP70, HSP60 (chaperonins), HSP40 (DNAJ-class proteins), and HSP20 (small heat shock proteins) [9]. Within each subfamily, some members are constitutively expressed, inducible regulated, and/or targeted to different compartments [7,10,11]. Genes encoding HSPs are also transcriptionally regulated by a variety of physiologic processes not typically associated with cell stress, including cell cycle, cell proliferation, and differentiation [12].

Aberrant expression of HSPs has been reported in a wide range of human tumors, including breast, endometrial, ovarian, colon, lung, and prostate [13,14]. In cancer cells, the HSPs network is extensively remodeled and could participate in the functional metabolism of tumor cells, protect them from harmful factors, provide an immunogenic context and allow tumor cells to tolerate genetic alterations, which would otherwise be fatal [15,16,17]. The expression of HSPs has been associated with tumor cell proliferation and differentiation, as well as with resistance to apoptosis and poor prognosis [18]. These observations, while intriguing, have not resolved whether the association between cancer evolution and HSPs is causal or correlative.

Here, we aimed to more precisely estimate the relationship between HSP expression and prognosis in breast cancer, and highlight the functional implications of their deregulation by using a multi-omics integrative analysis. 

An in silico analysis on differential expression of HSP family members in breast and other tumors was firstly performed, and the differentially expressed members (50 up-regulated and 26 down-regulated) were checked for genetic alterations and promoter methylation status. The prognostic value of HSP members and association with clinical variables were also assessed. Next, to highlight the important biological pathways deregulated as a consequence of HSPs expression, the lists of HSPs co-expressed genes and miRNAs extracted by different cross-platforms were used to perform functional analyses. Finally, proteomics investigations on a large sample set of breast cancer tissues showed additional complexity of the HSPs network in BC, with different protein isoforms identified for each HSP gene, probably performing additional functions and/or interactions. Our findings identified two distinct HSP-clusters with opposite functions in breast cancer: The first one with 19 members, was significantly associated with a poor prognosis and affected both cell cycle and DNA replication. The second HSP-cluster included 10 members associated with good prognosis, presumably affecting receptor signaling and Epithelial to Mesenchymal Transition (ETM). Interestingly, the HSPs-network involved also several miRNAs and protein isoforms that, in turn, could add new perspectives on HSPs contribution to BC. To the best of our knowledge, this is the first attempt exploring the potential mechanisms of the HSPs-mRNAs-miRNAs-proteins network, underlining that HSP-based regulation of BC progression is a multi-level process. We propose that the identified HSP-clusters can be used to stratify patients according to their predicted clinical outcome.

## 2. Materials and Methods

### 2.1. Expression Analysis of HSP Members Using UALCAN and Oncomine

The mRNA expression levels of each HSP member in tumors and their normal tissue counterparts were analyzed using UALCAN (https://ualcan.path.uab.edu/, accessed date 26 February 2021) and Oncomine database (https://www.oncomine.org/resource/login.html, accessed date 26 February 2021). UALCAN is a comprehensive, user-friendly, and interactive web resource for analyzing cancer OMICS data (TCGA, MET500, and CPTAC) [19]. For each query, the database provides graphs and plots depicting the expression profile of protein and miRNAs between normal and cancer tissues and evaluates epigenetic regulation of gene expression by promoter methylation. The expression level of HSP members was normalized as transcript per million reads, and a *p*-value of no more than 0.01 calculated through the Student’s *t*-test was considered to be significant. Oncomine, an online microarray database, can analyze the mRNA expression differences between tumor and normal tissues in 20 different human cancers. The thresholds were set as follows: *p*-value: 0.01; fold change: 2; gene rank: 10%; analysis type: cancer vs. normal analysis; data type: mRNA. 

### 2.2. Genetic Alterations and Epigenetic Regulation of HSP Members Using cBioPortal and UALCAN 

The frequency of HSPs alterations (amplification, deep deletion, and missense mutations) in BC patients was assessed using the OncoPrint tool of cBioPortal (http://www.cbioportal.org, accessed date 26 February 2021). cBioportal is an interactive open-source platform, that provides large-scale cancer genomics data sets database [20]. Promoter methylation status was analyzed using UALCAN. 

### 2.3. Evaluation of the Prognostic Value of HSP Members Using KM Plotter Database and Human Protein Atlas

The correlation between the mRNA expression levels of HSP members and the survival probability of BC patients was analyzed using the Kaplan-Meier Plotter database (http://kmplot.com/analysis/, accessed date 26 February 2021) [21]. For each HSP gene, Distant Metastasis Free Survival (DMFS), Overall Survival (OS), and Relapse Free Survival were assessed. The Affimetrix ID probes were entered into the database by using the multigene classifier. Patients were divided into high and low expression groups by using the best cutoff of mRNA expression. The database provided statistical plots for individual genes. The correlation between HSPs protein expression levels via immunohistochemistry analysis and overall survival of BC patients was analyzed using the Human Protein Atlas (https://www.proteinatlas.org/, accessed date 26 February 2021) [22]. For each HSP protein, the immunohistochemical staining was evaluated and scored based on staining intensity (negative, weak, moderate, or strong) and the fraction of stained cells (<25%, 25~75%, >75%). The staining quantity of each protein via IHC was determined as the percentage of stained cells in 10 high-power fields. All annotation data and immunohistochemistry images analyzed in the present investigation and all anti-body validation data are publicly available at https://www.proteinatlas.org/, accessed date 26 February 2021.

### 2.4. Relationship between HSP Members and Clinical-Pathological Parameters Using Bc-GenExMiner and GOBO

The correlation between the mRNA expression of HSP members and different clinical-pathological parameters, such as ER/PR/HER receptor status and lymph node (N) involvement, were evaluated using the Breast cancer Gene-Expression Miner v4.5 database (bcGenExMiner v4.5) [23], an on-line statistical mining tool (http://bcgenex.ico.unicancer.fr, accessed date 26 February 2021) of published annotated BC transcriptomic data (DNA microarrays [*n* = 10,716] and RNA-seq [*n* = 4712]). bcGenExMiner offers the possibility to explore gene expression, prognostic and correlation analyses, providing various kinds of plots. The association between HSP members and grading (G1/G2/G3) was performed by Gene expression-based Outcome for Breast cancer Online (GOBO database) [24]. GOBO (http://co.bmc.lu.se/gobo, accessed date 26 February 2021) enables a rapid assessment of gene expression levels, the identification of co-expressed genes and association with the outcome for single genes, gene sets, or gene signatures in an 1881-sample breast cancer data set, generated on Affymetrix U133A microarrays. 

### 2.5. Analysis of HSPs Co-Expressed Genes and Pathways Enrichment Analysis 

The co-expression profiles of the HSPs associated with poor and good prognosis were retrieved using three different online platforms, namely UALCAN (https://ualc an.path.uab.edu/, accessed date 26 February 2021), GOBO (http://co.bmc.lu.se/gobo/coexpressed_genes.pl, accessed date 26 February 2021) and bcGenExMiner (http://bcgenex.centregauducheau.fr/BC-GEM/GEM-Requete.php?mode=5, accessed date 26 February 2021). Results were statistically analyzed using Pearson’s correlation coefficient (*p* ≥ 0.4 and *p* ≤ −0.4). Positive and negative HSPs co-expressed genes were properly sorted to obtain two lists of up-regulated genes in poor prognosis (positive co-expressed genes for HSPs correlated with poor prognosis and negative co-expressed genes for HSPs correlated with good prognosis) and up-regulated in good prognosis (positive co-expressed genes for HSPs correlated with good prognosis and negative co-expressed genes for HSPs correlated with poor prognosis). Each gene list was submitted into the FunRich database [25], (http://www.funrich.org/, accessed date 26 February 2021) a stand-alone software tool used mainly for functional enrichment and interaction network analysis of genes and miRNA. Enriched biological pathways were ranked by *p*-value and the top six significant pathways were exhibited as bar charts. A *p*-value < 0.05 was regarded as statistically significant. 

### 2.6. Interactome Construction Using STRING Database

STRING (https://string-db.org, accessed date 26 February 2021) is a database of known and predicted protein-protein interactions. The interactions include direct (physical) and indirect (functional) associations derived from computational prediction, knowledge transfer between organism, and interactions aggregated from other (primary) databases analysis [26].

### 2.7. HSP-Mediated Regulatory Network of miRNAs-mRNAs 

The list of HSPs co-expressed miRNAs was retrieved using bcGenExMiner. Pearson’s correlation coefficient was considered significant when *p* ≥ 0.4 and *p* ≤ −0.4. Positive and negative HSPs co-expressed miRNAs were properly sorted to obtain two lists of up-regulated miRNAs in a poor and in good prognosis. Within each list, only the miRNAs recurring at least three times were selected for miRNAs-mRNAs network construction using miRNet (www.mirnet.ca, accessed date 26 February 2021), an easy-to-use web-based tool designed for creation, customization, visual exploration, and functional interpretation of miRNA-target interaction networks [27]. miRNet was also used to predict miRNAs acting on HSP-cluster associated with poor and good prognosis. 

### 2.8. Identification of HSP Proteins and Isoforms in Breast Cancer Tissues 

Two-dimensional (2D)-IPG electrophoresis was performed on 63 protein extracts from breast cancer tissues obtained following surgical interventions during the years 2004–2010 at the “La Maddalena” Hospital of Palermo, as previously described [28,29,30,31,32,33]. Briefly, the surgical samples homogenated overnight at 4 °C with RIPA buffer, were centrifugated, and the supernatants were dialyzed against ultrapure distilled water, lyophilized and resuspended in ISOT buffer. Aliquots of 45 μg of total proteins were rehydrated in rehydration buffer containing 8 M urea, 2% CHAPS, 10 mM DTE, and 0.5% carrier ampholytes (Resolyte 3.5–10). The electrophoretic separation was performed on 18 cm long strips with a pH range of 3–10. The strips were then equilibrated in a solution containing 50 mM Tris-HCl pH 6.8, 6 M urea, 0.5% SDS, 30% Glycerol, 130 mM DTE and 135 mM Iodoacetamide and then separated on 9–16% linear gradient polyacrylamide gels (SDS-PAGE), with a constant current of 20 mA/gel, as previously described [34,35,36]. The gels were silver stained and analyzed with the dedicated Image-Master 2D Platinum software. Proteins of interest were excised from the gel and the identity was assigned by peptide mass fingerprinting using the Voyager DE MALDI-TOF mass spectrometer as described [37,38,39,40,41,42]. The expression level of protein spots was calculated as the volume of the spots (i.e., integration of optical density over the spot area), relative to the sum of the volume of all spots on each gel (% Vol). Measurements of relative expression levels of individual protein spots were normalized in each proteomic map for actin content (N % V), as previously reported [43]. 

## 3. Results

We systematically analyzed by data mining 95 heat shock genes (and the protein members that they encode), grouped into 6 sub-families (Table 1): (1) HSP 110 (HSPH) with 2 genes; (2) HSP90 (HSPC) with 4 genes; (3) HSP70 (HSPA) with 15 genes; (4) HSP60 (Chaperonins) with 14 genes; (5) HSP20 (HSPB), with 11 genes; (6) HSP40 (DNAJ) with 49 genes.

### 3.1. Gene Expression Analysis of HSP Family Members between Normal and Cancer Tissues 

**Figure 1:**
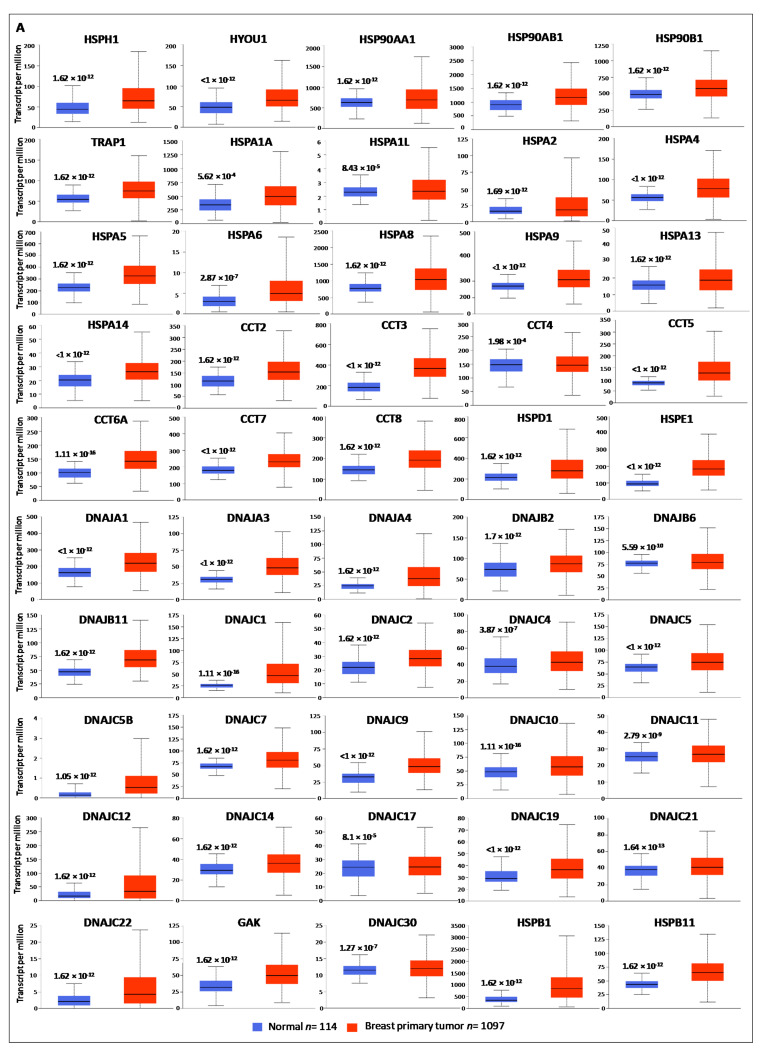

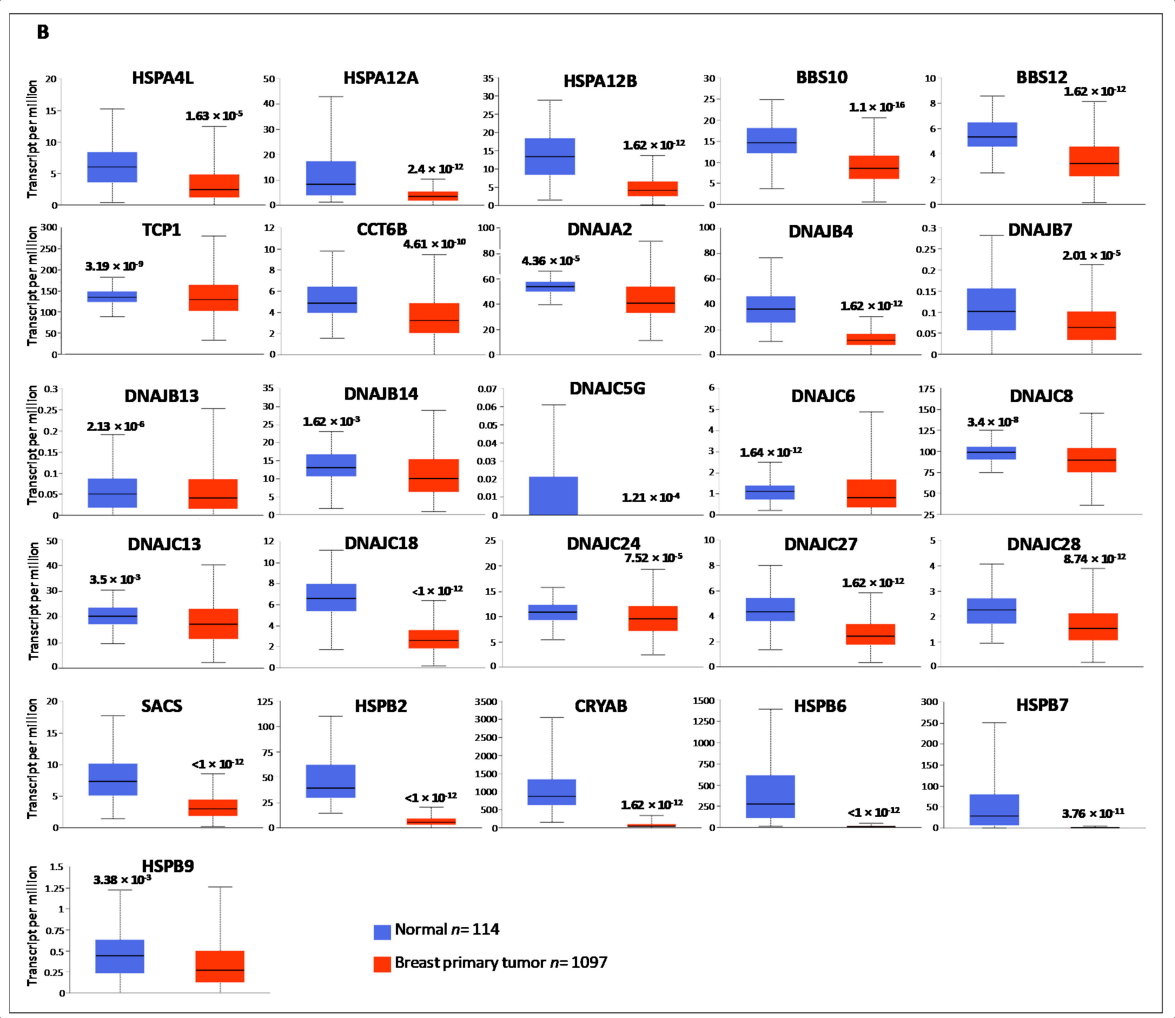
Gene expression analysis of HSP members between normal and breast cancer samples analyzed by UALCAN database. (**A**) Box plots of HSP genes up-regulated in BC compared to normal tissues. (**B**) Box plots of HSP genes down-regulated in BC compared to normal tissues. *p* < 0.01 was considered significant.

The expression profiles of HSP family members in breast cancer were determined using UALCAN database. Among the 95 HSP members, 76 were significantly deregulated in cancer tissues when compared to the corresponding normal tissues. In particular, 50 HSPs, were found up-regulated (Figure 1A, Table 2) while 26 HSPs, were found down-regulated in breast cancer than normal tissues (Figure 1B, Table 2). Interestingly, the number of transcripts, per million, was spanning between 0.07 for DNAJA5G and 3500 for HSPB1 and CRYAB, suggesting that some members are expressed at very low levels (HSPA1L, HSPB9, DNAJB7, DNAJ5B, DNAJC6, DNAJC27, and DNAJC28) while others are expressed at high levels (HSPA1A, HSPA8, HSPB6, HSP90AA1, HSP90AB1, and HSP90B1). Next, Oncomine meta-analysis was employed to assess the comprehensive expression of HSP members across 20 cancer types (Figure S1). The database compared the HSPs gene expression levels in cancer versus normal samples across a wide variety of datasets in different cancer types. A total of 1460 analyses showed a significant statistical difference for mRNA expression in cancer versus normal samples (the selected thresholds for the analyses were: *p*-value: 1 × 10^−4^; fold change: 2; gene rank: top 10%; data type: mRNA; sample type: clinical specimens). Overlapping results were recorded between UALCAN and Oncomine platforms except for HSPA1A, HSPA1L, HSPA2, DNAJA4, DNAJB2, DNAJC4, DNAJC11, DNAJC21 and GAK found up-regulated in breast cancer but collectively down-regulated in the other tumors, and DNAJC8, DNAJC24, SACS and HSPB6 down-regulated in breast cancer and collectively up-regulated in other tumors. The obtained results clearly indicated that the HSP members are involved in carcinogenesis, acting both as oncogenes or suppressor genes, and suggested that some of them could exert specific roles in different cancer types. Moreover, a high agreement was obtained using different bioinformatics platforms, supporting the robustness of the results.

### 3.2. Genetic and Epigenetic Alterations of HSP Members 

**Figure 2:**
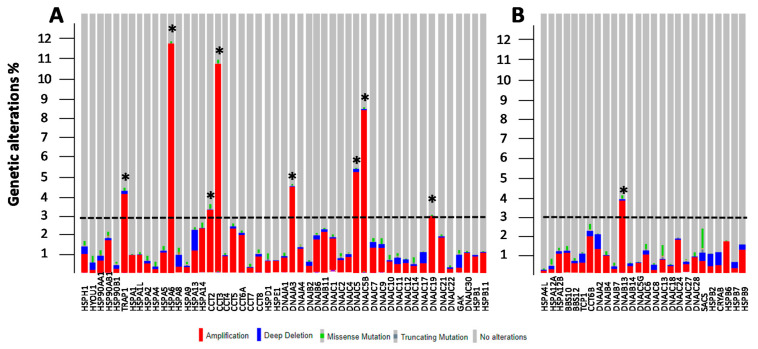
Analysis of genetic alterations in HSP members differentially expressed in BC. The percentage of alterations in HSP genes was extracted by using the OncoPrint tool of cBioPortal containing sequencing data of 2509 patients. Red and blue represent amplification, and deep deletion, respectively, green and grey represent missense and truncating mutations. Asterisk * indicates the corresponding HSP members showing a percentage of alteration higher than 3%. Histograms showing the percentage of genetic alterations of HSP genes up-regulated (**A**), and down-regulated (**B**) in BC.

**Figure 3:**
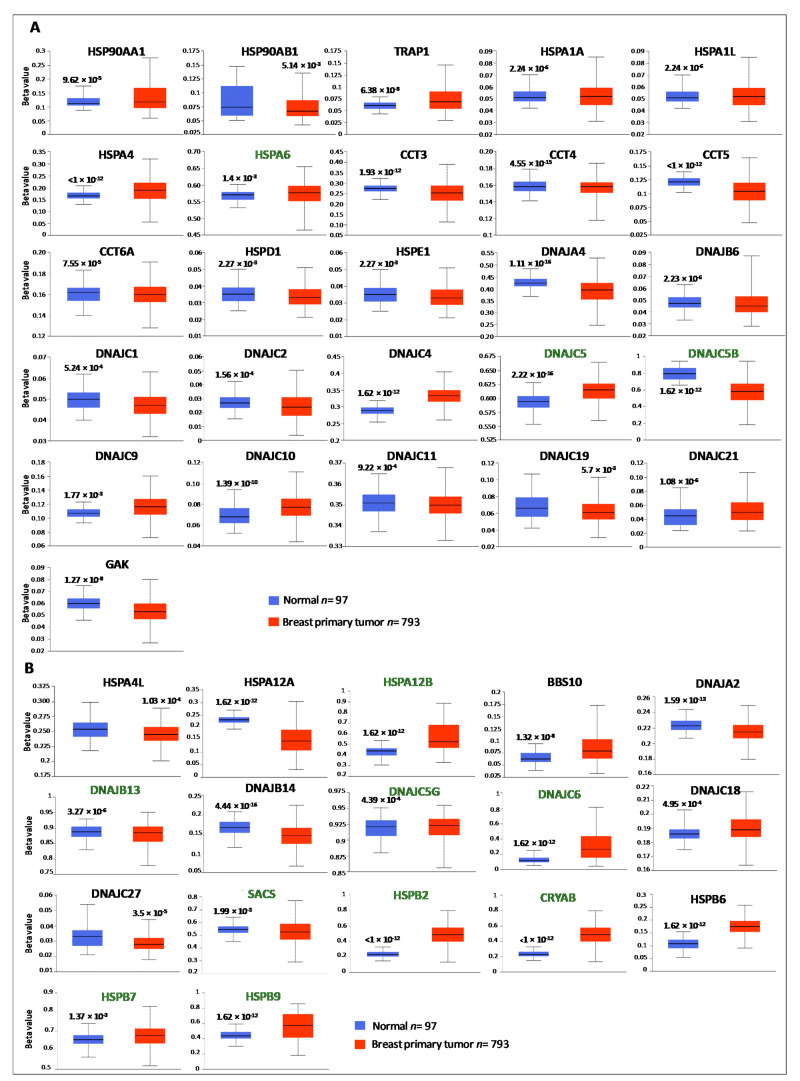
Analysis of promoter methylation status in HSP members differentially expressed in BC. For each HSP member the levels of promoter methylation between normal and breast cancer were retrieved from UALCAN database. Beta value cut-off indicates hyper-methylation [beta value: 0.7–0.5] or hypo-methylation [beta-value: 0.3–0.25]. HSP members with hyper-methylated promoters are marked in green; HSP members with hypo-methylated promoters are marked in black. Box plots showing the promoter methylation status between normal and breast primary tumors in HSPs up-regulated (**A**) and down-regulated in BC (**B**). *p* < 0.01 was considered significant.

It is well-recognized that the expression levels of selected genes could depend on genetic (amplifications or deletions) and epigenetic (promotor methylation status) alterations. To verify whether the deregulated expression of HSP members in breast cancer could be caused by one or a combination of these factors, the genetic mutations of HSP members, in a cohort of breast cancer patients using cBioPortal web, were analyzed. The mutation rate was higher for the HSP members up-regulated in BC (Figure 2A), spanning from 0.4% to 12%. Among these, TRAP1, HSPA6, CCT2, CCT3, DNAJA3, DNAJC5, DNAJC5B and DNAJC19 showed a percentage of alterations higher than 3%. A lower mutation rate was recorded for HSP members down-regulated in BC (Figure 2B), spanning from 0.2% to 4%. Among these, only DNAJB13 showed a percentage of alteration higher than 3%. Concerning the type of HSPs alteration, amplification was the major one, followed by deletion. A very low percentage of missense mutations was detected in all the analyzed samples. Promoter methylation status was analyzed from TCGA data through UALCAN database. Among the differentially expressed HSPs, 26 over 50 up-regulated (Figure 3A) and 17 over 26 down-regulated (Figure 3B), showed statistical differences in promoter methylation status between normal and breast cancer samples. Different beta value cut-off indicates hyper-methylation [beta value: 0.7–0.5] or hypo-methylation [beta-value: 0.3–0.25]. Interestingly, based on beta values, 23/26 HSPs were found with the hypo-methylated promoter and 9/17 HSPs were validated with the hyper-methylated promoter. According to expression pattern analysis among the HSPs up-regulated in BC, 15/26 showed lower promoter methylation while among the HSPs down-regulated in BC, 10/17 showed higher promoter methylation in tumor samples compared with normal samples. These data suggested that the deregulated HSPs gene expression associated with BC may be partly due to genetic and epigenetic alterations.

### 3.3. HSPs Expression and Clinical Outcome

**Figure 4:**
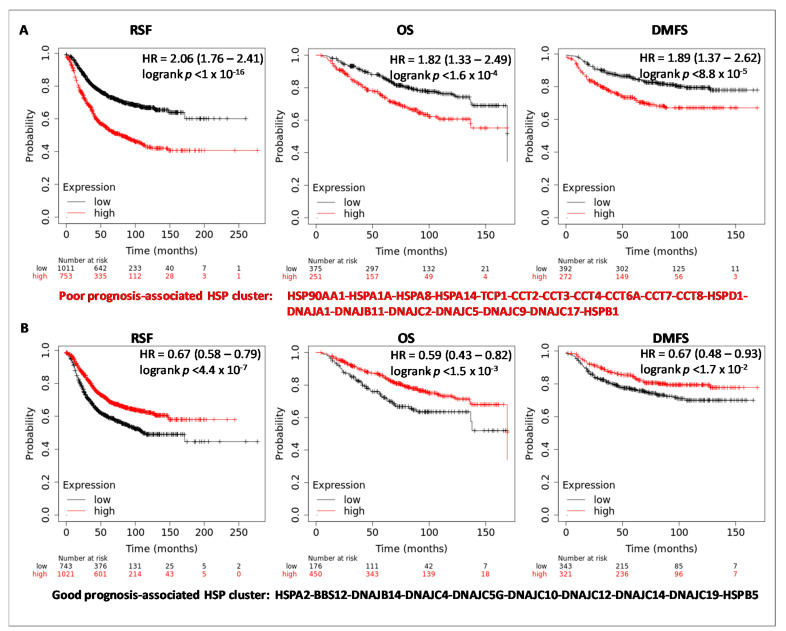
Survival curves of HSP members significantly associated with prognosis. The analysis is shown for Relapse Free Survival (RFS) Overall Survival (OS) and Distant Metastasis Free Survival (DMFS) using the Kaplan-Meier Plotter database. Patients were divided into two groups by using the best cut-off of probe expression. Survival curves of HSP-cluster associated with poor (**A**) and good (**B**) prognosis.

To analyze the prognostic value of the HSP members, Kaplan Meier-plotter database restricted to BC was searched. For each HSP gene (using the Affimetrix ID probe listed in Table 1), the survival outcomes were evaluated as Relapse Free Survival (RSF), Distant Metastasis Free Survival (DMFS), and Overall Survival (OS), (Table S1). Patients were divided into two groups (high and low) based on the expression levels of individual HSPs by selecting the best cut-off. In this case, the software computed all possible cut-off values between the lower and the upper quartiles and the best performing threshold was used as the cut-off value. HSPs expression levels were considered as significantly associated with prognosis when all survival outcomes (RSF, DMSF, and OS) were coherently significant (Table 3, Figure S2). The increased expression levels of 21 HSPs were significantly associated with a worse prognosis, whereas high transcriptional levels of 13 HSPs favored a good prognosis. Among these, only HSPA1B, DNAJB1, DNAJB8, DNAJB12, and DNAJC16 were not differentially expressed in BC, and thus, not further considered. Interestingly, when the survival analyses were re-evaluated, including the panel of worse prognosis-associated HSPs, better prognostic values than the individual ones were obtained (Figure 4A). Similar results (Figure 4B) were obtained including the panel of good prognosis-associated HSPs. These results revealed that collectively, the selected clusters of 19 HSPs and 10 HSPs could be represent robust biomarkers for poor and good prognosis in breast cancer, than individual members.

### 3.4. Relationship between HSP Members and Clinical-Pathological Parameters

The relationship between the expression levels of HSP-clusters differentially associated with prognosis and current clinical-pathological parameters was assessed using bcGeneXMiner and GOBO databases (Table 4). Clinical parameters included immuno-cytochemical expression of estrogen receptor (ER), progesterone receptor (ER), human epidermal growth receptor 2 (HER), lymph node (N) metastases, and grading (G1-G2-G3). The HSP-cluster associated with poor prognosis showed significant correlations with ER-/PR-, HER+, N+, and G3 tumors, which clinically identify more aggressive tumors. On the contrary, for the HSP-cluster associated with a good prognosis, an inverse trend of correlation with ER+/PR+, HER- N- and G1 tumors was found. Accordingly, it is well known that HER-, N- and G1 tumors are less aggressive. The obtained results confirm that the identified clusters can group patients with homogeneous clinical characteristics and underline their robustness in predicting prognosis in subgroups of patients.

### 3.5. HSPs-Gene Co-Expression Networks 

**Figure 5:**
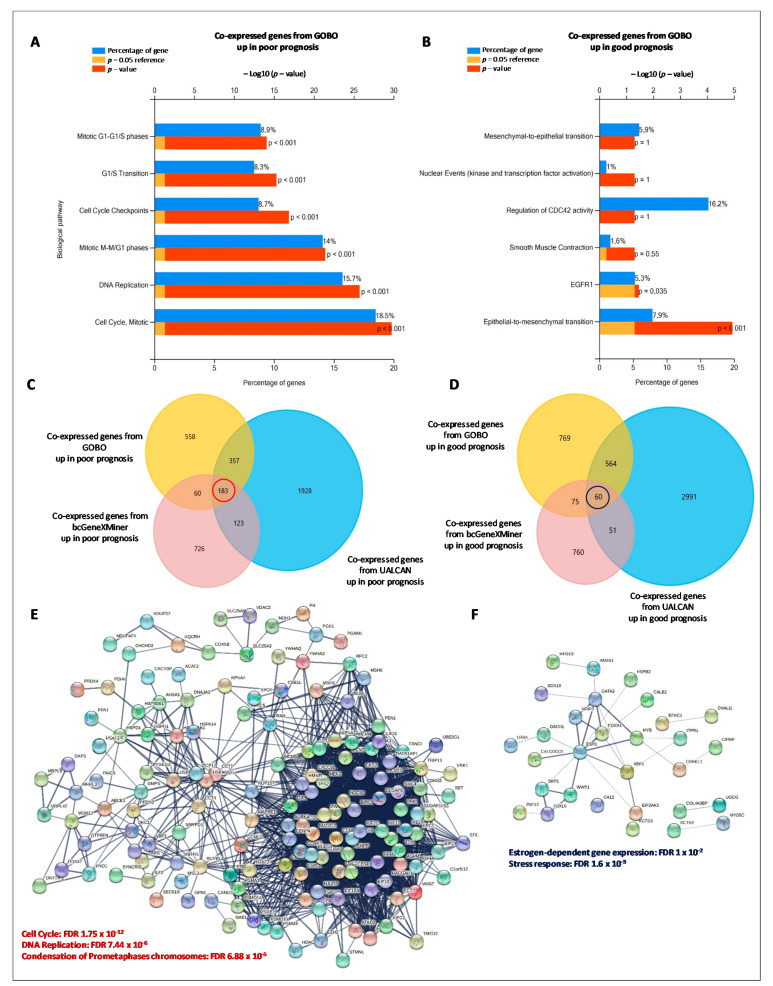
Enrichment pathway analysis of HSPs co-expressed genes significantly correlated with prognosis. Significantly enriched biological pathways were ranked by *p*-value using the FunRich 3.0 software. (**A**,**B**) The top six biological pathways in which the HSPs co-expressed genes up-regulated in poor and good prognosis, derived from GOBO, were significantly involved. (**C**,**D**) Venn diagrams of overlapping HSPs co-expressed genes up-regulated in poor and good prognosis, derived from GOBO, UALCAN, and bc-GenExMiner. (**E**,**F**) Protein-protein interaction network of the common HSPs co-expressed genes derived from the three cross-platforms GOBO, UALCAN and bc-GenExMiner using STRING database. The network was performed with medium confidence (combined score > 0.4) and disconnected nodes were deleted.

To unveil the mechanisms by which HSPs deregulation affect breast cancer development and influence prognosis, we identified the predicted genes that are transcriptionally co-expressed with individual HSPs via computational analysis. The co-expressed genes for the HSP-cluster that are significantly associated with a poor and good prognosis were queried to GOBO, UALCAN, and bc-GenExMiner database, respectively, using a Pearson correlation score of ≥ 0.4. The lists of the derived positive and negative HSPs co-expressed genes (Table S2), were appropriately sorted to obtain up-regulated genes in poor prognosis and up-regulated genes in good prognosis. Functional enrichments in the HSP-regulated networks (biological pathways) were highlighted by using the Fun Rich tool. Although each HSP was significantly associated with different genes in different databases, they were implicated in similar biological functions. Overall, biological processes were included in the mitotic cell cycle, DNA replication and cell cycle checkpoint for the co-expressed HSP genes up-regulated in poor prognosis, independently from the used database (Figure 5A, Figure S3A), suggesting that these HSPs-regulated biological processes might play a significant role in the initiation and progression of breast cancer. Less robust results were obtained for the co-expressed HSP genes up-regulated in good prognosis (Figure 5B, Figure S3B): the biological processes in which these genes are involved were Epithelial to Mesenchymal Transition (ETM) for the gene list derived from GOBO and bc-GenExMiner, and receptor signaling network (PAR-1, c-MET, VEGFR1, VEGFR2, HER2) for the gene list derived from UALCAN. Interestingly, when the list of common genes from the three bioinformatics platforms (Figure 5C,D) was used to find functional protein-protein interactions by using the STRING analysis tool (Figure 5E,F), a significant network, involved in cell cycle and DNA replication, was obtained for the co-expressed HSP genes up-regulated in poor prognosis. Again, the protein-protein interaction network derived from the co-expressed HSP genes up-regulated in good prognosis, was less robust and affected the Estrogen-dependent gene expression. Collectively, the pathway analysis of HSP co-expressed genes showed a specific signature for poor prognosis, affecting cell cycle and DNA replication. 

### 3.6. Regulatory Network Analysis of HSPs-miRNA-mRNA 

To further explore the molecular mechanisms responsible for HSPs effects on BC, we analyzed the networks of HSPs co-expressed miRNAs generated by the bc-GenExMiner database. Positive and negative HSPs co-expressed miRNAs were properly sorted to obtain two lists of up-regulated miRNAs in poor and in good prognosis. Only the miRNAs repeated at least three times were selected for miRNA-mRNA network construction using miRNet. A total of 14 miRNAs were clustered within the miRNAs network up-regulated in poor prognosis (Table 5). Interestingly, the target genes were involved in the cell cycle (Figure S4A). A total of 20 miRNAs were clustered within the miRNAs network up-regulated in good prognosis (Table 5). The target genes were implicated in the negative regulation of DNA-dependent transcription (Figure S4B). Interestingly, among the HSPs co-expressed miRNAs up-regulated in poor prognosis 5 miRNAs (hsa-mir-320b-2, hsa-mir-545, hsa-mir-651, hsa-mir-570, hsa-mir-643) were significantly up-regulated in breast cancer than normal tissues, as verified by using UALCAN, while among the HSPs associated miRNAs up-regulated in good prognosis, 10 miRNAs (hsa-mir-143, hsa-mir-196b, hsa-mir-145, hsa-mir-150, hsa-mir-1228, hsa-mir-1910, hsa-mir-1247, hsa-mir-411, hsa-mir-370, hsa-mir-770) were significantly down-regulated in BC compared to normal tissues. As the mRNAs-miRNAs network was created considering the HSPs clusters associated with prognosis, a greater interconnection mRNAs-miRNAs was recorded for the cluster associated with poor prognosis (Figure S4C), compared to the network obtained for HSPs-cluster associated with good prognosis (Figure S4D). As expected, collectively they were involved in protein folding. Several miRNAs, selected as key components of the networks, showed significant differences between breast cancer than normal tissues. In particular, 12 of them were up-regulated in breast cancer tissues, while 6 of them were down-regulated in breast cancer. These results, for the first time, indicated that the HSPs deregulation in BC affected epigenetic networks involving also several microRNAs, pointing that the complex network triggered by HSPs deregulation provides a distinctive molecular portrait of each tumor.

### 3.7. Proteomics Expression of HSP Members and Prognostic Significance

**Figure 6:**
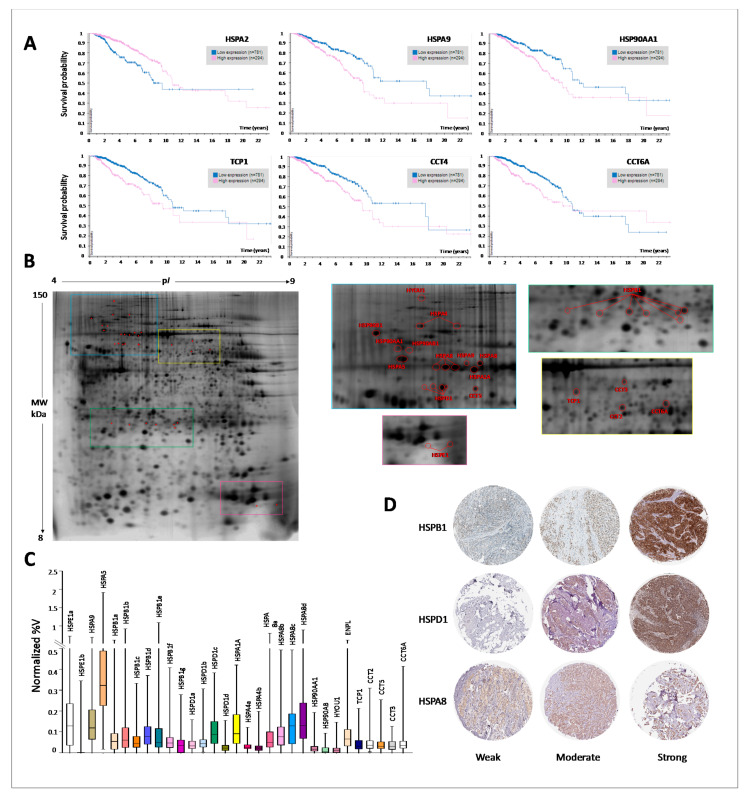
Proteomic expression of HSP members and prognostic significance. (**A**) Survival analysis of HSPs protein expression evaluated by immunohistochemistry (IHC) staining, using The Human Protein Atlas (HPA) database. The database was queried for each HSP member, and only significant analyses were reported; (**B**) Representative proteomic map of a breast cancer tissue. The 2-D separation was performed on IPG gel strips (18 cm, 3.0–10 NL) followed by the SDS-PAGE on a vertical linear-gradient slab gel (9–16%T). HSP protein members identified by Maldi-Tof spectrometry are marked with the Gene name. When present, different isoforms of the same protein were jointly labeled; (**C**) Quantitative analysis of HSP proteins, given as box-plot graph, in breast cancer tissues. Quantification was deduced by the 2D gels, analyzed by Image-Master software. In the ordinate are the values of N%V; (**D**) Representative IHC images for individual HSP proteins (HSPB1, HSPD1, and HSPA8) in breast cancer tissues showing weak, moderate and strong staining, respectively retrieved by the HPA database. The antibody immune reactivity was observed in several cell compartments, such as cytoplasm, plasma membrane as well as in nucleus.

Complementing the transcriptomics results with proteomics ones allows studying globally the HSPs-network within cancer cells and is also necessary to validate the existence of different protein isoforms as well as the exact localization of proteins, tightly linked to their function. The prognostic value of HSPs protein expression, evaluated by immunohistochemistry (IHC) staining, was retrieved using The Human Protein Atlas (HPA) database (Figure 6A). High expression levels of 5 HSP members (HSPA9, HSP90AA1, TCP1, CCT4 and CCT6A) were significant associated with shorter overall survival, while a high expression level of HSPA2 was associated with a good prognosis. Moreover, taking advantage of our previous proteomic data, we performed a comprehensive screening of protein spots, in different proteomic maps, and collectively the following HSP protein members were identified: HYOU1 (HYOU1), HSP90AA1 (HS90A), HSP90AB (HS90B), HSP90B1 (ENPL), HSPA1A (HS71A), HSPA4 (HSP74, 2 isoforms), HSPA5 (BIP), HSPA8 (HSP7C, 4 isoforms), HSPA9 (GRP75), HSPD1 (CH60, 4 isoforms), TCP1 (TCPA), CCT2 (TCPB), CCT3 (TCPG), CCT5 (TCPE), CCT6A (TCPZ), HSPB1 (HSP27, 7 isoforms), HSPE1 (CH10, 2 isoforms). Figure 6B shows a representative proteomic map of a breast surgical tissue silver-stained, with cropped windows where the 17 identified HSPs proteins and 14 isoforms are marked with labels corresponding to the gene name. The protein identity was assessed by Maldi-Tof (Table S3). Different isoforms of the same protein were labeled by alphabetic letters starting from the more acidic one. Several HSP proteins and isoforms showed high variability of expression between analyzed patients (Figure 6C), suggesting that different proteins and isoforms should be used for patient stratification. Finally, the immunohistochemical analysis using the HPA database gives us the possibility to define HSPs protein localization in different compartments at a single-cell and subcellular level and also provided important spatial information in the context of neighboring cells. Figure 6D reports immunohistochemical staining of BC tumors showing weak, moderate and strong expression of selected HSPs, based on the major number of identified isoforms in proteomics maps. Interestingly, antibody immunoreactivity was observed in several cellular compartments, such as cytoplasm, plasma membrane, and nucleus. Collectively, our proteomic data on HSPs expression and prognostic value are in line with genomic data. Although, proteomic investigations showed a more complex scenario in which different HSP-isoforms can operate within the cancer cells, creating new functional arrangements, difficult to predict based on gene expression. In the hope for the clinical use of HSP-clusters, both the gene and protein expression levels should be taken into consideration, for a more accurate prognosis. 

## 4. Discussion 

HSPs are a ubiquitous and highly conserved protein class, essential for cell protection from a wide range of harmful conditions, including heat shock, oxidative stress, inflammation, and proteotoxic stress (a specific form of deleterious stress causing increased levels of misfolded proteins). However, when the cells reach the limit of stress tolerance, they activate apoptosis or autophagy. HSPs play critical roles in inhibiting pro-apoptotic molecules through the modulation of several signaling cascades such as JNK, AKT, and NF-κB [44]. Several HSP members are closely associated with the onset and progression of several human cancer types [45,46,47,48,49], but the expression pattern and potential roles of HSPs in breast cancer, as well as their underlying regulatory mechanisms, remain poorly investigated. Only two recent studies investigated the prognostic significance of HSP members in BC, exclusively limited to the transcriptomic analysis [50,51]. Here, for the first time, we performed an integrated multi-omics analysis to assess the prognostic value and the functional implications of HSPs deregulation in BC. Using UALCAN database, we found that collectively HSP family members were deregulated in BC. In particular, 50 HSPs members were significantly up-regulated in cancer compared to normal tissues, while 26 HSPs members were down-regulated. These results indicated that HSPs could act both as oncogenes or tumor suppressor genes. Moreover, the Oncomine analysis of HSPs deregulation in different cancer types showed that HSPs exert both, pro- and anti-tumorigenic actions depending on the tumor type. 

HSP members are coded by distinct chromosomal loci that are not clustered. The molecular basis of HSPs deregulation in BC has not been well-studied and may have multiple molecular etiologies. For example, associated with genetic alterations and/or epigenetic modifications. Collectively, we showed that the mutation rate (amplification followed by deletion) was higher in HSP members up-regulated in BC, while a lower mutation rate was detected in HSP members down-regulated in BC. Besides, HSPs promoter regions were found to be commonly hypo-methylated in HSP members up-regulated in BC, while a general tendency to hyper-methylated promoters was detected in HSP members down-regulated in BC. In alignment with our results, the CpG island located in the promoter regions of the DNAJC10 gene was found to be frequently hypo-methylated in breast cell lines [52]. We speculate that genetic alterations and epigenetic deregulations could in part explain the deregulation of HSPs in BC. Other mechanisms responsible for HSPs deregulation are: (1) The heat shock factor (HSF) family that ensures prompt transcriptional activation of HSP members under stress and equally precipitous switch-off after recovery [53]; (2) the genetic changes associated with tumor progression, producing over-expressed onco-proteins, mostly mutated and/or conformationally altered proteins, may elicit an HSPs response; (3) the aneuploidy (defined as abnormal chromosome number), and its associated abnormalities [54], such as chromosomal instability (CIN), could impair HSPs expression and function. Aneuploidy alters the relative dosage of genes on the affected chromosomes, leading to cellular stress response at least partially due to impaired protein folding capacity. This, in turn, affects the cellular protein quality control pathways that preserve homeostasis and induces chronic proteotoxic stress (broadly referring to the overburdening of cellular systems that maintain proper protein folding and homeostasis) [55,56]. Consequently, the aneuploid cells would exhibit an over-production of proteins, relative to the chaperone systems needed to fold nascent polypeptides or the degradation systems that remove misfolded or damaged proteins. This is of particular importance regarding the maintenance of the correct stoichiometry of macromolecular complexes, whose subunits may be encoded by genes on different chromosomes. Further studies are needed to more comprehensively explore the detailed molecular mechanisms of altered HSPs expression in BC.

The prognostic analysis, performed on HSPs gene expression levels and clinical outcome, identified two HSP-clusters, associated with a poor and good prognosis, respectively, in line with previous reports [50,51]. Analytically, when the HSP subfamilies were considered, the chaperonins subfamily (TCP1, CCT2, CCT3, CCT4, CCT6A, CCT7, and CCT8) was collectively associated with poor prognosis. Accordingly, the expression levels of different CCT members are reported as up-regulated in various cancers [57]. In particular, it was estimated that CCT members facilitate the folding of approximately 10% of newly synthesized proteins [58], and in cancer cells they could potentially fold more proteins, with substrates, including oncogenic proteins and mediators of oncogenesis [59]. A more heterogeneous involvement with prognosis was detected for HSP40 members, with 7 members significantly associated with poor prognosis (DNAJA1, DNAJB1, DNAJB11, DNAJC2, DNAJC5, DNAJC9, DNAJC17) and 10 members associated with good prognosis (DNAJB8, DNAJB12, DNAJB14, DNAJC4, DNAJC5G, DNAJC10, DNAJC12, DNAJC14, DNAJB16, and DNAJC19). HSP40 members acting as co-chaperones regulate the major functions of other HSPs (HSP90, HSP70, and HSP60) and could be able to switch the canonical functions of HSPs towards oncogenic roles. HSP40 members work as tumor suppressors and inhibit tumor growth [60]. Other members have been implicated in cancer development and metastasis, such as DNAJA1 (in glioblastoma and prostate), DNAJB11 (in ovarian tumors), and DNAJC9 (cervical) [61].

Interestingly, the HSP-cluster associated with poor prognosis showed significant correlations with ER-/PR-/HER+, lymph node metastases, and higher grading, identifying more aggressive tumors. On the contrary, the HSPs cluster associated with a good prognosis showed a significant correlation with ER+/PR+, HER-, lymph node-negative, and lower grading tumors. Collectively, these results confirm the robustness of the identified HSP clusters in predicting prognosis in clinical subgroups of patients. 

To explore HSPs-related pathways altered in BC, genes co-expressed with HSPs were analyzed. We found that HSPs co-expressed genes associated with poor prognosis were enriched with cell cycle, DNA replication and cell cycle checkpoint, commonly oncogenic processes associated with cell proliferation. Therefore, we hypothesize that HSP-networks, affecting cell cycle, and DNA replication, may provide a robust measure of proliferation rate and more aggressive clinical course in BC. Less robust results were obtained for the HSPs co-expressed genes up-regulated in a good prognosis. The pathways enrichment analysis of these genes showed that they are involved in ETM, receptors signaling (PAR-1, c-MET, VEGFR1, VEGFR2, HER2) and Estrogen-dependent gene expression and thus further functional investigations are needed to validate these findings.

It is well-known that miRNA can act as master players at any stage of breast cancer development by targeting multiple mRNAs that are implicated in tumor suppressor or oncogenic signaling pathways [62]. To gain new functional insights into the molecular mechanisms underlying the role of HSPs in BC tumorigenesis, we screened the biological roles of candidate miRNAs involved in HSPs-regulated networks. In these networks, 14 potential miRNAs, clustered within the miRNA network up-regulated in poor prognosis, were identified as candidates’ hub miRNAs (hsa-mir-320b-2, hsa-mir-545, hsa-mir-941-2, hsa-mir-651, hsa-mir-383, hsa-mir-1914, hsa-mir-570, hsa-mir-647, hsa-mir-1227, hsa-mir-593, hsa-mir-300, hsa-mir-643, hsa-mir-626, hsa-mir-1289-1) and, accordingly with the HSP-co-expressed gene functions, the target genes were implicated in the cell cycle. A total of 20 miRNAs, clustered within the miRNA network up-regulated in good prognosis, were identified as possible hub miRNAs (hsa-mir-23a, hsa-mir-143, hsa-mir-185, hsa-mir-196b, hsa-mir-145, hsa-mir-150, hsa-mir-1228, hsa-mir-1910, hsa-mir-450a-1, hsa-mir-548i-1, hsa-mir-1247, hsa-mir-411, hsa-mir-1299, hsa-mir-370, hsa-mir-544a, hsa-mir-1200, hsa-mir-3654, hsa-mir-770, hsa-mir-555, hsa-mir-604), and the target genes were implicated in the negative regulation of transcription, DNA dependent. Several of the identified miRNAs were also differentially expressed in breast cancer than in normal tissues, emphasizing the important role of these miRNAs in breast carcinogenesis and progression, as already reported by several authors [63,64,65,66,67,68,69,70]. We surmise that miRNAs, related to a worse prognosis, may exert their oncogenic functions by silencing breast onco-suppressors, while the miRNAs related to a better prognosis may exert protective roles by silencing breast oncogenes. Further research and validation experiments that explore the clinical and biological roles of selected miRNAs may yield a better understanding of the mechanisms underlying breast cancer growth, metastasis, and survival. However, in this study, for the first time, we showed that the HSPs deregulation in BC affected epigenetic networks involving also microRNAs.

As proteins are considered the molecular effectors, the characterization of HSPs protein expression profiles represents the best chance of better understanding their molecular context-dependent functions. Proteomic data on HSPs expression and prognostic value are in line with the transcriptomic results, although proteomic investigations showed a more complex scenario in which different HSP-proteoforms (probably arising from alternatively spliced RNA transcripts and post-translational modifications (PTM)) can operate within the cancer cells, creating new functional arrangements, difficult to predict on gene expression. Based on IHC analyses, only 5 HSP members (HSPA9, HSP90AA1, TCP1, CCT4 and CCT6A) were significant associated with shorter overall survival, while a higher expression level of HSPA2 was associated with a good prognosis. This stringency in the prognostic value of HSP proteins could partly depend on the expression of multiple proteoforms, probably localized in different subcellular compartments, affecting a variety of intracellular signaling pathways. It is well-known that the HSPs activity is regulated by PTM such as phosphorylation, acetylation, methylation, ubiquitination, glycosylation, S-nitrosylation [71]. Since the addition and reciprocal removal of chemical groups can be triggered very rapidly, PTMs provide an efficient switch to precisely regulate the HSPs functions and activities. For instance, the HSP27 (HSPB1) function is modulated by phosphorylation at different serine residues, catalyzed by the mitogen-activated protein kinase MAPK2 and 3 [72]. Moreover, in clinical cancer tissues, various phosphorylation patterns of HSP27 have been found to associate with the aggressiveness of tumor phenotype [73]. Consequently, it has been proposed that HSP27 phosphorylation isoforms could also represent useful tumor markers, while a more comprehensive analysis of tissue samples will be required. Besides, each HSP is properly localized in a specific cellular compartment(s). For instance, HSP70 and HSP90 can be localized in the cytosol and the nucleus, grp78 (HSPA5) in the endoplasmic reticulum. Whereas, HSP60 (HSPD1) is found in mitochondria [74]. Some HSPs can also be found on the cell surface, such as HSP60 and HSP70, especially in lipid rafts (plasma membrane subdomains, containing high levels of cholesterol and glycosphingolipids) [75]. More recently, HSPs are considered key players in the intercellular cross-talk [76]. They may be secreted through Extracellular Vesicles (EVs), e.g., exosomes, which derive from endosomes and multivesicular bodies. HSP27, HSP60, HSC70, HSP70, and HSP90 were found in the extracellular environment where their role is believed to be immunogenic and may induce either a pro- or anti-inflammatory response. For example, EV containing HSP70 on their surface activates macrophages and natural killer cells. HSPs can be released from cells in free, soluble form, possibly via Golgi. In this form, HSPs circulate via blood throughout the organism and act in an endocrine fashion: [77].

## 5. Conclusions

Altogether, based on bioinformatics analysis and experimental validation, we provided a reliable integrated analysis of the HSPs expression pattern, prognostic value, and potential regulatory mechanisms in breast cancer. HSPs represent a multitasking protein family exerting multi-level functions within BC cells. HSP network is dynamic within cancer cells whose biochemistry and function depend on cellular and environment milieu. In this intricate scenario, two different HSP-clusters here identified might serve as a potential panel for prognosis evaluation. The usefulness of obtained data should be considered prospectively because, in the era of personalized therapy, the molecular characterization of the two HSP-clusters should be given the possibility to stratify patients with different prognoses. Moreover, several clinical trials are currently investigating the use of HSP-inhibitors or HSP-based vaccines for cancer therapy and immunotherapy. We believe that, rather than a single HSP member, multiple HSPs should be targeted so that they have a better chance of reverting the clinical phenotype. In fact, the simultaneous deregulation of several HSPs in cancer cells could be orchestrating new interactive circuits that define tumor progression. However, further validations are needed to judge their comprehensive prognostic impact in BC.

## Figures and Tables

**Table 1:** List of 95 HSP family members classified into 6 sub-families according to their molecular weight (HSP110, HSP90, HSP70, HSP60, HSP40 and HSP20). For each HSP, the Gene name, Chromosomic localization, Affimetrix ID probe, Protein name, Uniprot database access number, Abbreviated protein name and Theoretical molecular weight, are reported.

HSP Family	Gene Name	Chromosomic Localization	Affimetrix ID	Protein Name	Uniprot Access (Abbreviated Name)	Molecular Weigth (Da)
HSP110 (HSPH)	HSPH1	13q12.3	235573_at	Heat shock 105 kDa/110 kDa protein 1	Q92598 (HS105)	96,865
	HYOU1	11q23.3	200825_s_at	Hypoxia up-regulated protein 1	Q9Y4L1 (HYOU1)	111,335
HSP90 (HSPC)	HSP90AA1	14q32.31	214328_s_at	Heat shock protein HSP 90-alpha	P07900 (HS90A)	84,660
	HSP90AB1	6Q21,1	200064_at	Heat shock protein HSP 90-beta	P08238 (HS90B)	83,264
	HSP90B1	12q23.3	200599_s_at	Endoplasmin	P14625 (ENPL)	92,469
	TRAP1	16P13.3	201391_at	Heat shock protein 75 kDa, mitochondrial	Q12931 (TRAP1)	80,110
HSP70 (HSPA)	HSPA1A	6p21.33	200799_at	Heat shock 70 kDa protein 1A	P0DMV8 (HS71A)	70,052
	HSPA1B	6p21.33	202581_at	Heat shock 70 kDa protein 1B	P0DMV9 (HS71B)	70,052
	HSPA1L	6p21.33	210189_at	Heat shock 70 kDa protein 1-like	P34931 (HS71L)	70,375
	HSPA2	14q23.3	211538_s_at	Heat shock-related 70 kDa protein 2	P54652 (HSP72)	70,021
	HSPA4	5q31.1	208815_x_at	Heat shock 70 kDa protein 4	P34932 (HSP74)	94,331
	HSPA4L	4q28	205543_at	Heat shock 70 kDa protein 4L	O95757 (HS74L)	94,512
	HSPA5	9q33.3	230031_at	Endoplasmic reticulum chaperone BiP	P11021 (BIP)	72,333
	HSPA6	1q23.3	117_at	Heat shock 70 kDa protein 6	P17066 (HSP76)	71,028
	HSPA7	1q23.3	-	Putative heat shock 70 kDa protein 7	P48741 (HSP77)	40,244
	HSPA8	11q24.1	221891_x_at	Heat shock cognate 71 kDa protein	P11142 (HSP7C)	70,898
	HSPA9	5q31.1	200690_at	Stress-70 protein, mitochondrial	P38646 (GRP75)	73,680
	HSPA12A	10q25.3	214434_at	Heat shock 70 kDa protein 12A	O43301 (HS12A)	74,978
	HSPA12B	20p13	229172_at	Heat shock 70 kDa protein 12B	Q96MM6 (HS12B)	75,688
	HSPA13	21q11.2	202557_at	Heat shock 70 kDa protein 13	P48723 (HSP13)	51,927
	HSPA14	10p13	226887_at	Heat shock 70 kDa protein 14	Q0VDF9 (HSP7E)	54,794
HSP60 (Chaperonins)	BBS10	12q21.2	219487_at	Bardet-Biedl syndrome 10 protein	Q8TAM1 (BBS10)	80,838
	BBS12	4q27	229603_at	Bardet-Biedl syndrome 12 protein	Q6ZW61 (BBS12)	79,085
	TCP1	6q25.3	222010_at	T-complex protein 1 subunit alpha	P17987 (TCPA)	60,344
	CCT2	12q15	201947_s_at	T-complex protein 1 subunit beta	P78371 (TCPB)	57,488
	CCT3	1q23	200910_at	T-complex protein 1 subunit gamma	P49368 (TCPG)	60,534
	CCT4	2p15	200877_at	T-complex protein 1 subunit delta	P50991 (TCPD)	57,924
	CCT5	5p15.2	229068_at	T-complex protein 1 subunit epsilon	P48643 (TCPE)	59,671
	CCT6A	7p11.2	201326_at	T-complex protein 1 subunit zeta	P40227 (TCPZ)	58,024
	CCT6B	17q12	206587_at	T-complex protein 1 subunit zeta-2	Q92526 (TCPW)	57,821
	CCT7	2p13.2	200812_at	T-complex protein 1 subunit eta	Q99832 (TCPH)	59,367
	CCT8	21q22.11	200873_s_at	T-complex protein 1 subunit theta	P50990 (TCPQ)	59,621
	HSPD1	2q33.1	200807_s_at	60 kDa heat shock protein, mitochondrial	P10809 (CH60)	61,055
	HSPE1	2q33.1	205133_s_at	10 kDa heat shock protein, mitochondrial	P61604 (CH10)	10,932
	MKKS	20p12	218138_at	McKusick-Kaufman/Bardet-Biedl syndromes putative chaperonin	Q9NPJ1 (MKKS)	62,342
HSP40 (DNAJ)	DNAJA1	9p21.1	200881_s_at	DnaJ homolog subfamily A member 1	P31689 (DNJA1)	44,868
	DNAJA2	16q12.1	226994_at	DnaJ homolog subfamily A member 2	O60884 (DNJA2)	45,746
	DNAJA3	16p13.3	205963_s_at	DnaJ homolog subfamily A member 3, mitochondrial	Q96EY1 (DNJA3)	52,489
	DNAJA4	15q25.1	225061_at	DnaJ homolog subfamily A member 4	Q8WW22 (DNJA4)	44,798
	DNAJB1	19p13.2	200666_s_at	DnaJ homolog subfamily B member 1	P25685 (DNJB1)	38,044
	DNAJB2	2q35	202500_at	DnaJ homolog subfamily B member 2	P25686 (DNJB2)	35,580
	DNAJB3	2q37.1	-	DnaJ homolog subfamily B member 3	Q8WWF6 (DNJB3)	16,559
	DNAJB4	1p31.1	203810_at	DnaJ homolog subfamily B member 4	Q9UDY4 (DNJB4)	37,807
	DNAJB5	9p13.1	212817_at	DnaJ homolog subfamily B member 5	O75953 (DNJB5)	39,133
	DNAJB6	7q36.3	209015_s_at	DnaJ homolog subfamily B member 6	O75190 (DNJB6)	36,087
	DNAJB7	22q13.2	1552675_at	DnaJ homolog subfamily B member 7	Q7Z6W7 (DNJB7)	35,434
	DNAJB8	3q21.3	237284_at	DnaJ homolog subfamily B member 8	Q8NHS0 (DNJB8)	25,686
	DNAJB9	7q31.1	202843_at	DnaJ homolog subfamily B member 9	Q9UBS3 (DNJB9)	25,518
	DNAJB11	3q27.3	223054_at	DnaJ homolog subfamily B member 11	Q9UBS4 (DJB11)	40,514
	DNAJB12	10q22.1	202866_at	DnaJ homolog subfamily B member 12	Q9NXW2 (DJB12)	41,860
	DNAJB13	11q13.4	230936_at	DnaJ homolog subfamily B member 13	P59910 (DJB13)	36,118
	DNAJB14	4q23	226399_at	DnaJ homolog subfamily B member 14	Q8TBM8 (DJB14)	42,516
	DNAJC1	10p12.31	218409_at	DnaJ homolog subfamily C member 1	Q96KC8 (DNJC1)	63,883
	DNAJC2	7q22.1	213097_s_at	DnaJ homolog subfamily C member 2	Q99543 (DNJC2)	71,996
	DNAJC3	13q32.1	225284_at	DnaJ homolog subfamily C member 3	Q13217 (DNJC3)	57,580
	DNAJC4	11q13.1	228622_s_at	DnaJ homolog subfamily C member 4	Q9NNZ3 (DNJC4)	27,593
	DNAJC5	20q13.33	224611_s_at	DnaJ homolog subfamily C member 5	Q9H3Z4 (DNJC5)	22,149
	DNAJC5B	8q13.1	232798_at	DnaJ homolog subfamily C member 5B	Q9UF47 (DNJ5B)	22,496
	DNAJC5G	2p23.3	1552450_a_at	DnaJ homolog subfamily C member 5G	Q8N7S2 (DNJ5G)	21,433
	DNAJC6	1p31.3	204721_s_at	Putative tyrosine-protein phosphatase auxilin	O75061 (AUXI)	99,997
	DNAJC7	17q21.2	202416_at	DnaJ homolog subfamily C member 7	Q99615 (DNJC7)	56,441
	DNAJC8	1p35.3	212490_at	DnaJ homolog subfamily C member 8	O75937 (DNJC8)	29,842
	DNAJC9	10q22.2	213088_s_at	DnaJ homolog subfamily C member 9	Q8WXX5 (DNJC9)	29,910
	DNAJC10	2q32.1	229588_at	DnaJ homolog subfamily C member 10	Q8IXB1 (DJC10)	91,080
	DNAJC11	1p36.31	215792_s_at	DnaJ homolog subfamily C member 11	Q9NVH1 (DJC11)	63,278
	DNAJC12	10q21.3	223722_at	DnaJ homolog subfamily C member 12	Q9UKB3 (DJC12)	23,415
	DNAJC13	3q22.1	212467_at	DnaJ homolog subfamily C member 13	O75165 (DJC13)	254,415
	DNAJC14	12q13.2	223420_at	DnaJ homolog subfamily C member 14	Q6Y2X3 (DJC14)	78,569
	DNAJC15	13q14.11	227808_at	DnaJ homolog subfamily C member 15	Q9Y5T4 (DJC15)	16,383
	DNAJC16	1p36.21	212911_at	DnaJ homolog subfamily C member 16	Q9Y2G8 (DJC16)	90,591
	DNAJC17	15q15.1	219861_at	DnaJ homolog subfamily C member 17	Q9NVM6 (DJC17)	34,687
	DNAJC18	5q31.2	238115_at	DnaJ homolog subfamily C member 18	Q9H819 (DJC18)	41,551
	DNAJC19	3q26.33	225358_at	Mitochondrial import inner membrane translocase subunit TIM14	Q96DA6 (TIM14)	12,499
	HSCB	22q12.1	223647_x_at	Iron-sulfur cluster co-chaperone protein HscB	Q8IWL3 (HSC20)	27,422
	DNAJC21	5p13.1	230893_at	DnaJ homolog subfamily C member 21	Q5F1R6 (DJC21)	62,028
	DNAJC22	12q13.12	220441_at	DnaJ homolog subfamily C member 22	Q8N4W6 (DJC22)	38,086
	SEC63	6q21	229969_at	Translocation protein SEC63 homolog	Q9UGP8 (SEC63)	87,997
	DNAJC24	11p13	213853_at	DnaJ homolog subfamily C member 24	Q6P3W2 (DJC24)	17,139
	DNAJC25	9q31.3	226859_at	DnaJ homolog subfamily C member 25	Q9H1X3 (DJC25)	42,404
	GAK	4p16.3	40225_at	Cyclin-G-associated kinase	O14976 (GAK)	143,191
	DNAJC27	2p23.3	227859_at	DnaJ homolog subfamily C member 27	Q9NZQ0 (DJC27)	30,855
	DNAJC28	21q22.11	220372_at	DnaJ homolog subfamily C member 28	Q9NX36 (DJC28)	45,806
	SACS	13q12.12	213262_at	Sacsin	Q9NZJ4 (SACS)	521,126
	DNAJC30	7q11.23	223367_at	DnaJ homolog subfamily C member 30, mitochondrial	Q96LL9 (DJC30)	25,961
HSP20 (HSPB)	HSPB1	7q11.23	201841_s_at	Heat shock protein beta-1	P04792 (HSPB1)	22,783
	HSPB2	11q23.1	205824_at	Heat shock protein beta-2	Q16082 (HSPB2)	20,233
	HSPB3	5q11.2	206375_s_at	Heat shock protein beta-3	Q12988 (HSPB3)	16,966
	CRYAA	21q22.3	210199_at	Alpha-crystallin A chain	P02489 (CRYAA)	19,909
	CRYAB	11q22.3	209283_at	Alpha-crystallin B chain	P02511 (CRYAB)	20,159
	HSPB6	19q13.12	226304_at	Heat shock protein beta-6	O14558 (HSPB6)	17,136
	HSPB7	1p36.13	218934_s_at	Heat shock protein beta-7	Q9UBY9 (HSPB7)	18,611
	HSPB8	12q24.23	221667_s_at	Heat shock protein beta-8	Q9UJY1 (HSPB8)	21,604
	HSPB9	17q21.2	230510_at	Heat shock protein beta-9	Q9BQS6 (HSPB9)	17,486
	OFD1	8q22.3	203569_s_at	Oral-facial-digital syndrome 1 protein	O75665 (OFD1)	116,671
	HSPB11	1p32	215691_x_at	Intraflagellar transport protein 25 homolog	Q9Y547 (IFT25)	16,297

**Table 2:** List of HSP members significantly up and down-regulated in BC analyzed in UALCAN database.

List of HSPs Up-Regulated in BC	UALCAN Gene Expression between Normal and Cancer Tissues (*p* Value)	List of HSPs Up-Regulated in BC	UALCAN Gene Expression between Normal and Cancer Tissues (*p* Value)	List of HSPs Down-Regulated in BC	UALCAN Gene Expression between Normal and Cancer Tissues (*p* Value)
HSPH1	1.62 × 10^−12^	HSPB1	1.62 × 10^−12^	HSPA4L	1.63 × 10^−5^
HYOU1	<1 × 10^−12^	HSPB11	1.62 × 10^−12^	HSPA12A	2.24 × 10^−12^
HSP90AA1	1.62 × 10^−12^	DNAJA1	<1 × 10^−12^	HSPA12B	1.62 × 10^−12^
HSP90AB1	1.62 × 10^−12^	DNAJA3	<1 × 10^−12^	BBS10	1.1 × 10^−16^
HSP90B1	1.62 × 10^−12^	DNAJA4	1.62 × 10^−12^	BBS12	1.62 × 10^−12^
TRAP1	1.62 × 10^−12^	DNAJB2	1.7 × 10^−12^	TCP1	3.19 × 10^−9^
HSPA1A	5.62 × 10^−4^	DNAJB6	5.59 × 10^−10^	CCT6B	4.61 × 10^−10^
HSPA1L	8.43 × 10^−5^	DNAJB11	1.62 × 10^−12^	HSPB2	<1 × 10^−12^
HSPA2	1.69 × 10^−12^	DNAJC1	1.11 × 10^−16^	CRYAB	1.62 × 10^−12^
HSPA4	<1 × 10^−12^	DNAJC2	1.62 × 10^−12^	HSPB6	<1 × 10^−12^
HSPA5	1.62 × 10^−12^	DNAJC4	3.87 × 10^−7^	HSPB7	3.76 × 10^−11^
HSPA6	2.87 × 10^−7^	DNAJC5	<1 × 10^−12^	HSPB9	3.89 × 10^−3^
HSPA8	1.62 × 10^−12^	DNAJC5B	1.05 × 10^−9^	DNAJA2	4.36 × 10^−5^
HSPA9	<1 × 10^−12^	DNAJC7	1.62 × 10^−12^	DNAJB4	1.62 × 10^−12^
HSPA13	1.62 × 10^−12^	DNAJC9	<1 × 10^−12^	DNAJB7	2.01 × 10^−5^
HSPA14	<1 × 10^−12^	DNAJC10	1.11 × 10^−16^	DNAJB13	2.13 × 10^−6^
CCT2	1.62 × 10^−12^	DNAJC11	2.97 × 10^−9^	DNAJB14	1.62 × 10^−3^
CCT3	<1 × 10^−12^	DNAJC12	1.62 × 10^−12^	DNAJC5G	1.21 × 10^−4^
CCT4	1.98 × 10^−4^	DNAJC14	1.62 × 10^−12^	DNAJC6	1.64 × 10^−12^
CCT5	<1 × 10^−12^	DNAJC17	8.1 × 10^−5^	DNAJC8	3.4 × 10^−8^
CCT6A	1.11 × 10^−16^	DNAJC19	<1 × 10^−12^	DNAJC13	3.5 × 10^−3^
CCT7	<1 × 10^−12^	DNAJC21	1.64 × 10^−13^	DNAJC18	<1 × 10^−12^
CCT8	1.62 × 10^−12^	DNAJC22	1.62 × 10^−12^	DNAJC24	7.52 × 10^−5^
HSPD1	1.62 × 10^−12^	GAK	1.62 × 10^−12^	DNAJC27	1.62 × 10^−12^
HSPE1	<1 × 10^−12^	DNAJC30	1.27 × 10^−7^	DNAJC28	8.74 × 10^−12^
				SACS	<1 × 10^−12^

**Table 3:** List of HSP members significantly associated with clinical outcome, including Relapse Free Survival (RFS), Overall Survival (OS), and Distant Metastasis Free Survival (DMFS), evaluated by KM Plotter database. The Log Rank value was marked in red when higher expression of selected HSP was associated with poor prognosis and in the black when higher expression of selected HSP was associated with a good prognosis. The probe expression value cut-off for each analysis is also reported.

Gene	RFS		OS		DMFS	
	Used Cut-Off	Logrank P	Used Cut-Off	Logrank P	Used Cut-Off	Logrank P
HSP90AA1	20,364	<1 × 10^−16^	23,504	5.7 × 10^−7^	20,054	3.8 × 10^−4^
HSPA1A	9077	9.4 × 10^−5^	13,346	2.5 × 10^−3^	9089	8 × 10^−4^
HSPA1B	2046	8.3 × 10^−4^	1254	9.2 × 10^−4^	1829	6.8 × 10^−3^
HSPA8	14,679	<1 × 10^−16^	14,679	5.5 × 10^−3^	14,018	1.7 × 10^−2^
HSPA14	490	1.7 × 10^−5^	477	1.7 × 10^−4^	490	2.2 × 10^−3^
TCP1	601	7.4 × 10^−11^	676	1.5 × 10^−7^	562	1.2 × 10^−4^
CCT2	5366	<1 × 10^−16^	5370	2.7 × 10^−8^	4520	2 × 10^−7^
CCT3	4554	2.9 × 10^−15^	4619	1.2 × 10^−2^	4735	1.9 × 10^−3^
CCT4	5934	1.10 × 10^−11^	5587	2.9 × 10^−2^	6778	3.32 × 10^−2^
CCT6A	1676	<1 × 10^−16^	1667	2 × 10^−8^	1678	7.6 × 10^−6^
CCT7	2498	<1 × 10^−16^	2394	5 × 10^−4^	2466	1.4 × 10^−3^
CCT8	5676	<1 × 10^−16^	4599	5.5 × 10^−5^	3613	1.1 × 10^−4^
HSPD1	11,874	<1 × 10^−16^	11,640	7.7 × 10^−7^	10,253	5.6 × 10^−6^
DNAJA1	5070	2 × 10^−14^	4099	2.7 × 10^−4^	4939	1 × 10^−4^
DNAJB1	2284	8.7 × 10^−4^	2312	4 × 10^−2^	2369	1.1 × 10^−2^
DNAJB11	1771	1.3 × 10^−9^	2300	4.20 × 10^−2^	1910	2.5 × 10^−4^
DNAJC2	1153	2.5 × 10^−16^	781	1.7 × 10^−3^	1049	9.1 × 10^−4^
DNAJC5	768	3.9 × 10^−2^	748	1.8 × 10^−3^	591	4.8 × 10^−2^
DNAJC9	1494	<1 × 10^−16^	1296	1.5 × 10^−7^	1328	1.3 × 10^−6^
DNAJC17	128	1.6 × 10^−2^	128	2 × 10^−2^	163	3.6 × 10^−3^
HSPB1	9981	2.4 × 10^−12^	5940	3.5 × 10^−4^	7584	1.3 × 10^−7^
HSPA2	536	2.2 × 10^−10^	624	5.8 × 10^−5^	563	1.7 × 10^−6^
BBS12	186	1 × 10^−10^	185	3.8 × 10^−4^	187	4.7 × 10^−3^
DNAJB8	26	4 × 10^−5^	23	3.7 × 10^−2^	24	7.4 × 10^−3^
DNAJB12	644	6.4 × 10^−10^	644	1.5 × 10^−2^	879	2.5 × 10^−2^
DNAJB14	757	5.5 × 10^−8^	1147	1.2 × 10^−4^	1164	2.7 × 10^−2^
DNAJC4	487	3.3 × 10−^10^	489	9 × 10^−3^	445	2.9 × 10^−3^
DNAJC5G	54	1.3 × 10^−8^	27	2.3 × 10^−3^	54	1.5 × 10^−2^
DNAJC10	171	5.3 × 10^−12^	202	9.6 × 10^−3^	187	4.3 × 10^−2^
DNAJC12	92	1.4 × 10^−7^	127	5.3 × 10^−4^	92	1.8 × 10^−5^
DNAJC14	433	3.8 × 10^−10^	393	5.2 × 10^−3^	529	1.5 × 10^−2^
DNAJC16	402	8.3 × 10^−11^	361	5.8 × 10^−9^	402	4.7 × 10^−8^
DNAJC19	562	1.4 × 10^−10^	578	1.6 × 10^−7^	578	3.1 × 10^−7^
CRYAB	575	1.7 × 10^−2^	522	3.8 × 10^−3^	848	3.2 × 10^−2^

**Table 4:** Relationship between HSPs mRNA expression in BC and clinical-pathological parameters, including estrogen receptor (ER), progesterone receptor (PR), and human epidermal growth receptor 2 (HER) status, lymph node (N) involvement, and grading (G1–G2–G3). Data were retrieved for each HSP using bcGeneXMiner and the grading was assessed using GOBO database. *p* < 0.05 was considered significant (*p* < 0.05 *, *p* < 0.01 **, *p* < 0.001 ***).

Gene Name	ER+/PR+	ER-/PR-	HER+	HER-	N+	N-	G1	G2	G3
HSPA1A	<0.0001 ***		-	-	0.0315 *		-	-	-
HSPA8		<0.0001 ***	0.0022 **		0.0002 ***		-	-	-
HSPA14		<0.0001 ***	<0.0001 ***		0.0134 **				<0.0001 ***
HSPB1	<0.0001 ***		-	-	<0.0001 ***			<0.0001 ***	
HSP90AA1		<0.0001 ***	<0.0001 ***		<0.0001 ***				<0.0001 ***
TCP1		<0.0001 ***	<0.0001 ***		0.0175 **				<0.0001 ***
CCT2	-	-	0.0066 **		0.0003 ***				<0.0001 ***
CCT3		<0.0001 ***	0.0001 ***		0.0025 **				<0.0001 ***
CCT4		<0.0001 ***	-	-	-	-			<0.0001 ***
CCT6A		<0.0001 ***	<0.0001 ***		<0.0001 ***				<0.0001 ***
CCT7		<0.0001 ***	-	-	<0.0001 ***				<0.0001 ***
CCT8		<0.0001 ***	-	-	-	-			<0.0001 ***
HSPD1		<0.0001 ***	<0.0001 ***		<0.0001 ***				<0.0001 ***
DNAJA1		<0.0001 ***	0.0091 **		<0.0001 ***				<0.0001 ***
DNAJB11		<0.0001 ***	<0.0001 ***		-	-	-	-	-
DNAJC2		<0.0001 ***	-	-	0.0002 ***				<0.0001 ***
DNAJC5		<0.0001 ***	<0.0001 ***		0.0050 **		-	-	-
DNAJC9		<0.0001 ***	0.0042 **		0.0213 *				<0.0001 ***
DNAJC17	<0.0001 ***		-	-	-	-	<0.0001 ***		
HSPA2	<0.0001 ***			<0.0001 ***	-	-	<0.0001 ***		
BBS12	<0.0001 ***			<0.0001 ***		0.0404 *	-	-	-
DNAJB14	<0.0001 ***		-	-	-	-		<0.0001 ***	
DNAJC4	0.01 *			0.0057 **		<0.0001 ***		<0.0001 ***	
DNAJC5G	-	-	-	-	-	-	-	-	-
DNAJC10	-	-	<0.0001 ***		-	-		0.00011 ***	
DNAJC12	<0.0001 ***			<0.0001 ***	-	-	<0.0001 ***		
DNAJC14	<0.0001 ***			0.0003 ***	-	-	-	-	-
DNAJC19	<0.0001 ***			<0.0001 ***	-	-	-	-	-
CRYAB		<0.0001 ***		<0.0001 ***	-	-	-	-	-

**Table 5:** List of the hubs miRNAs involved in the HSPs-miRNAs-mRNAs network. The red bolded miRNAs were significantly up-regulated in breast cancer than normal tissues; the black bolded miRNAs were significantly down-regulated in breast cancer than normal tissues. A *p*-value < 0.01 was regarded as significant.

HSPs Co-Expressed miRNAs Up-Regulated in Poor Prognosis		HSPs Co-Expressed miRNAs Up-Regulated in Good Prognosis	
**hsa-mir-320b-2**	**7.13 × 10^−7^**	hsa-mir-23a	-
**hsa-mir-545**	**4.38 × 10^−12^**	**hsa-mir-143**	**1.62 × 10^−12^**
hsa-mir-941-2	-	**hsa-mir-185**	**1.42 × 10^−4^**
**hsa-mir-651**	**<1 × 10^−12^**	**hsa-mir-196b**	**5.07 × 10^−2^**
**hsa-mir-383**	**5.48 × 10^−7^**	**hsa-mir-145**	**<1 × 10^−12^**
hsa-mir-1914	-	**hsa-mir-150**	**4.4 × 10^−11^**
**hsa-mir-570**	**5.56 × 10^−5^**	**hsa-mir-1228**	**3.04 × 10^−2^**
hsa-mir-647	-	**hsa-mir-1910**	**4.10 × 10^−5^**
hsa-mir-1227	-	hsa-mir-450a-1	-
hsa-mir-593	-	hsa-mir-548i-1	-
hsa-mir-300	-	**hsa-mir-1247**	**1.24 × 10^−14^**
**hsa-mir-643**	**9.17 × 10^−11^**	**hsa-mir-411**	**2.00 × 10^−5^**
hsa-mir-626	-	hsa-mir-1299	-
hsa-mir-1289-1	-	**hsa-mir-370**	**4.12 × 10^−5^**
		hsa-mir-544a	-
**HSPs-miRNAs network** **associated with poor prognosis**		hsa-mir-1200	-
		**hsa-mir-3654**	**8.15 × 10^−9^**
hsa-mir-16-5p	-	**hsa-mir-770**	**6.09 × 10^−05^**
hsa-mir-23b-3p	-	hsa-mir-555	-
**hsa-mir-15a-5p**	**3.88 × 10^−15^**	hsa-mir-604	-
hsa-mir-34a-5p	-		
hsa-mir-1-3p	-	**HSPs-miRNAs network associated with good prognosis**	
**hsa-mir-103a-3p**	**<1 × 10^−12^**		
**hsa-mir-148a-3p**	**6.5 × 10^−13^**	hsa-mir-16-5p	-
hsa-mir-424-5p	-	**hsa-mir-155-5p**	**1.62 × 10^−12^**
**hsa-mir-107**	**2.4 × 10^−11^**	**hsa-mir-146a-5p**	**5.77 × 10^−05^**
**hsa-mir-155-5p**	**1.62 × 10^−12^**	hsa-mir-124-3p	-
**hsa-mir-497-5p**	**<1 × 10^−12^**	hsa-mir-1-3p	-
**hsa-mir-181a-5p**	**8.31 × 10^−03^**	**hsa-mir-182-5p**	**1.62 × 10^−12^**
**hsa-mir-21-3p**	**<1 × 10^−12^**	**hsa-mir-191-5p**	**<1 × 10^−12^**
**hsa-mir-15b-3p**	**6.96 × 10^−10^**	**hsa-mir-17-5p**	**<1 × 10^−12^**
**hsa-mir-186-5p**	**0.00446**	hsa-mir-23b-3p	-
**hsa-mir-195-5p**	**<1 × 10^−12^**	hsa-mir-34a-5p	-
**hsa-mir-141-5p**	**1.62 × 10^−12^**	**hsa-mir-15a-5p**	**3.88 × 10^−15^**
**hsa-mir-29a-3p**	**1.96 × 10^−12^**	**hsa-mir-20a-5p**	**1.1 × 10^−11^**
hsa-mir-197-3p	-	**hsa-mir-195-5p**	**<1 × 10^−12^**
		hsa-mir-302c-3p	-

## Data Availability

Not applicable.

## Supplementary Materials

The following are available online at https://www.mdpi.com/2079-7737/10/3/247/s1, Figure S1: Gene expression analysis of HSP members in 20 different tumors and their corresponding normal tissues. Data were extracted for each HSP gene from ONCOMINE database. The selected thresholds for the analyses were: *p*-value: 1 × 10^−4^; fold change: 2; gene rank: top 10%; data type: mRNA; sample type: clinical specimens. The color depth was determined by the gene rank (top 1%–top 5%–top 10%). The number in the colored box represents the number of analyses meeting these thresholds. The red boxes indicate that the mRNA levels of target genes are higher in tumor tissues than in normal tissues, while blue boxes indicate that the mRNA levels of target genes are lower in tumor tissues than in normal tissues. (A) HSP110, HSP90 and HSP70 members; (B) HSP60 and HSP20 members; (C,D) HSP40 members. Table S1: Prognostic value of individual HSP members evaluated in KM plotter database. For each HSP (identified with an Affimetrix ID) survival outcome was evaluated as Relapse Free Survival (RSF), Distant Metastasis Free Survival (DMFS), and Overall Survival (OS). Patients were divided by the best cut-off vaue. Significant associations with prognosis are underlined in bold. The red color indicates that the higher HSP expression is significantly associated with a poor prognosis. The black color indicates that higher HSP expression is significantly associated with a good prognosis. Figure S2: Survival curves of HSP members significantly associated with poor (A–C) and good (D–F) prognosis. The analysis is shown for RFS (A–D) OS (B–E) and DMFS (C–F) using the Kaplan-Meier Plotter database. Patients were divided into two groups by using the best cut-off of probe expression. Table S2: Lists of positive and negative HSPs co-expressed genes derived by GOBO, UALCAN, and bc-GenExMiner database. Pearson correlation score was considered significant when ≥ 0.4 and ≤ −4. Figure S3. Enrichment pathway analysis of HSPs co-expressed genes significantly correlated with prognosis. Significantly enriched biological pathways were ranked by *p*-value using the FunRich 3.0 software. (A,B) The top six biological pathways in which the HSPs co-expressed genes up-regulated in poor and good prognosis, derived from UALCAN and bc-GenExMiner, were significantly involved. Figure S4. Construction of HSPs-miRNAs-mRNAs network and KEGG Pathway Enrichment Analysis using miRNet. (A) HSPs co-expressed miRNAs network up-regulated in poor prognosis; (B) HSPs co-expressed miRNAs network up-regulated in good prognosis; (C) HSPs-miRNAs network associated with poor prognosis; (D) HSPs-miRNAs network associated with good prognosis. In each network, yellow circles indicate the enriched genes retrieved by KEGG pathways analysis. The size of circles and squares indicates greater interconnection in the network (hubs miRNAs and genes). The hubs miRNAs involved in the network are listed and the expression of each miRNA in breast cancer tissues and normal controls was retrieved by UALCAN database. The red underlined miRNAs were significantly up-regulated in breast cancer than normal tissues; the black underlined miRNAs were significantly down-regulated in breast cancer than normal tissues. A *p*-value < 0.01 was regarded as significant. Table S3: List of the HSP proteins identified in the proteomics maps of breast cancer tissues by Maldi-Tof. For each protein the Abbreviated Name, the Uniprot AC, the Mowse Score, the Number of mass values matched, the Number of mass values searched, the % Masses Matched, the % Sequence Coverage, and the experimental MW (Da) and the p*I* are indicated.
