# Supplementary figures and images for: Prognostic and Functional Significant of Heat Shock Proteins (HSPs) in Breast Cancer Unveiled by Multi-Omics Approaches
Source: Biology (Basel). 2021 Mar 22;10(3):247. doi: 10.3390/biology10030247 (PMC8004706; doi:10.3390/biology10030247)

**A**

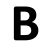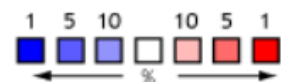

Figure S1

C

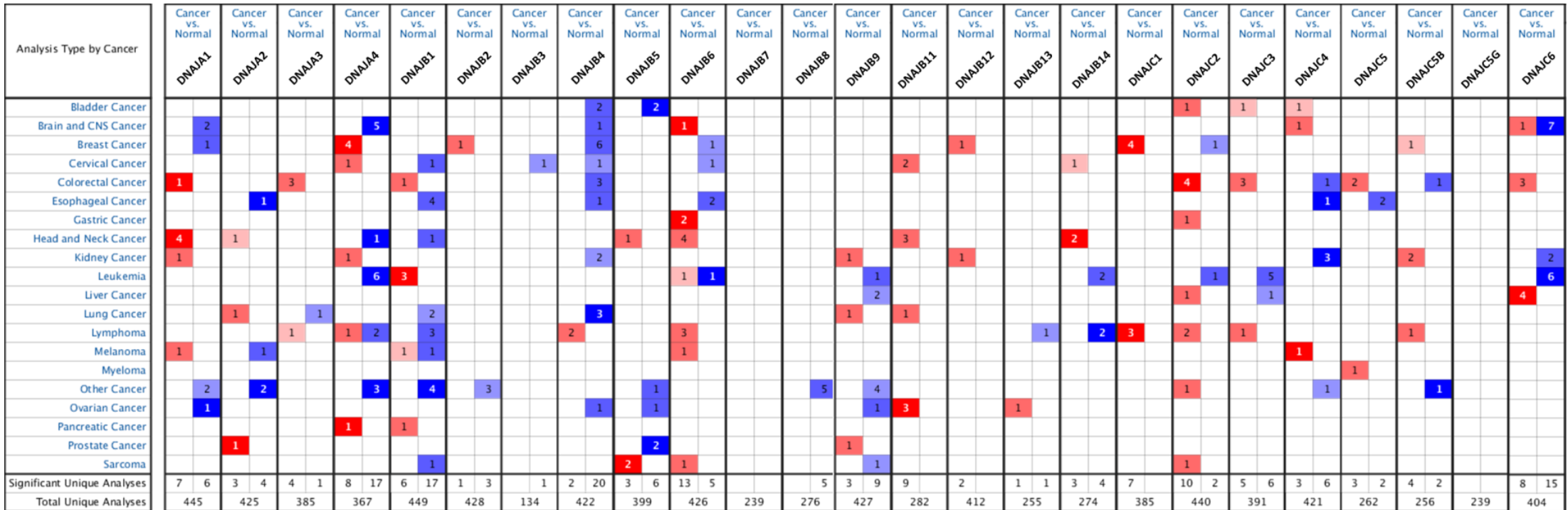

D

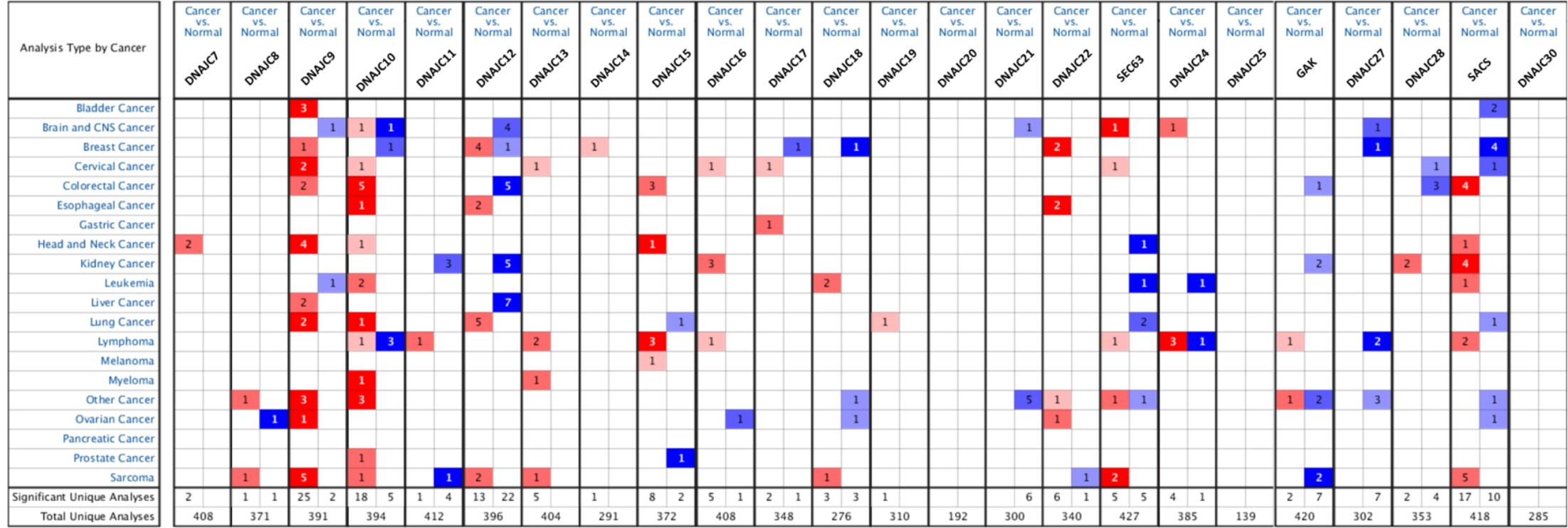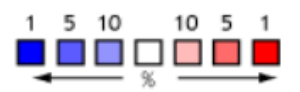

Supplement: Supplementary file 1 [file biology-10-00247-s001.zip › Supplementary/Figure S1.pdf]

Figure S2

A

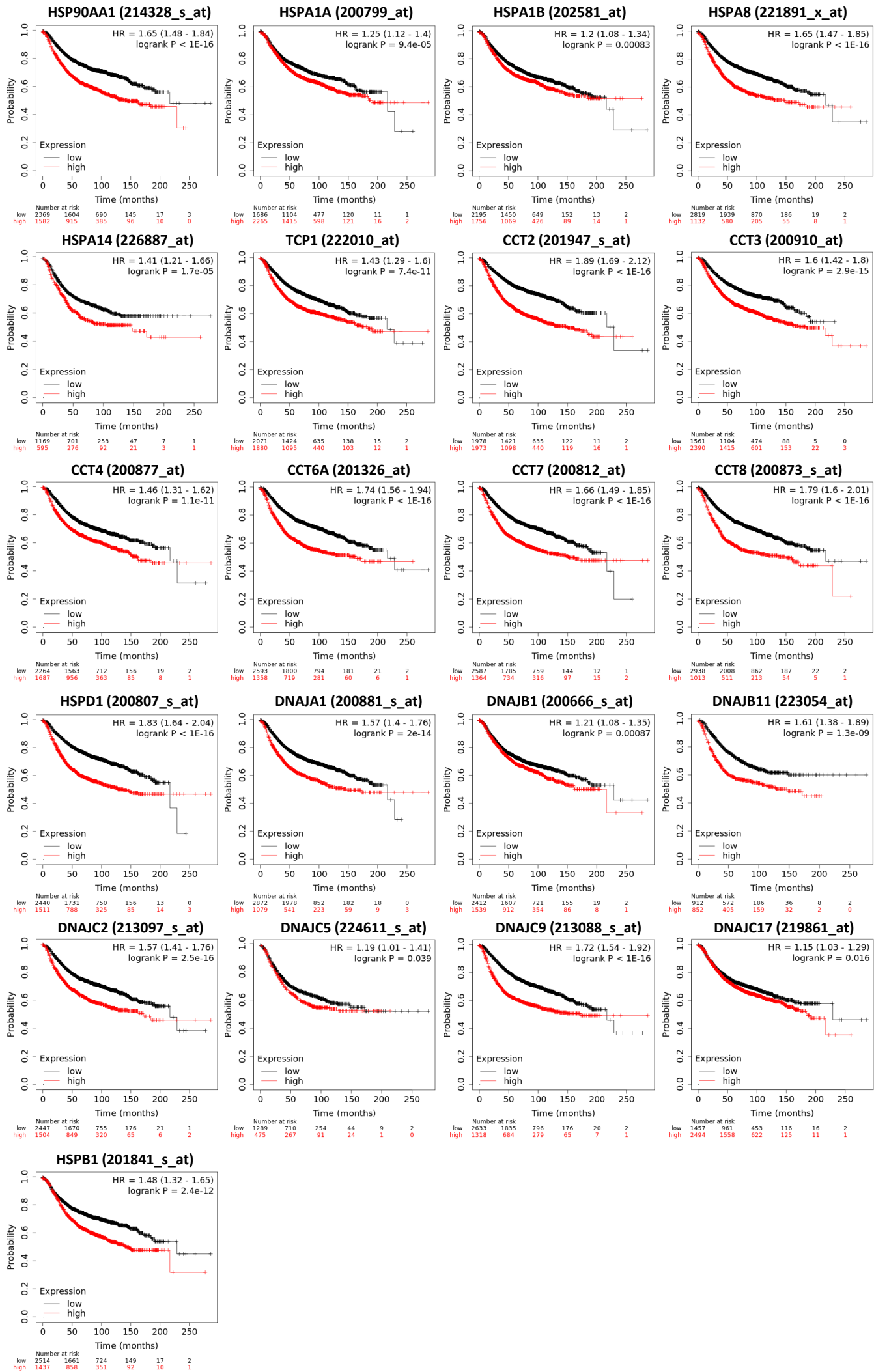

Figure S2

B

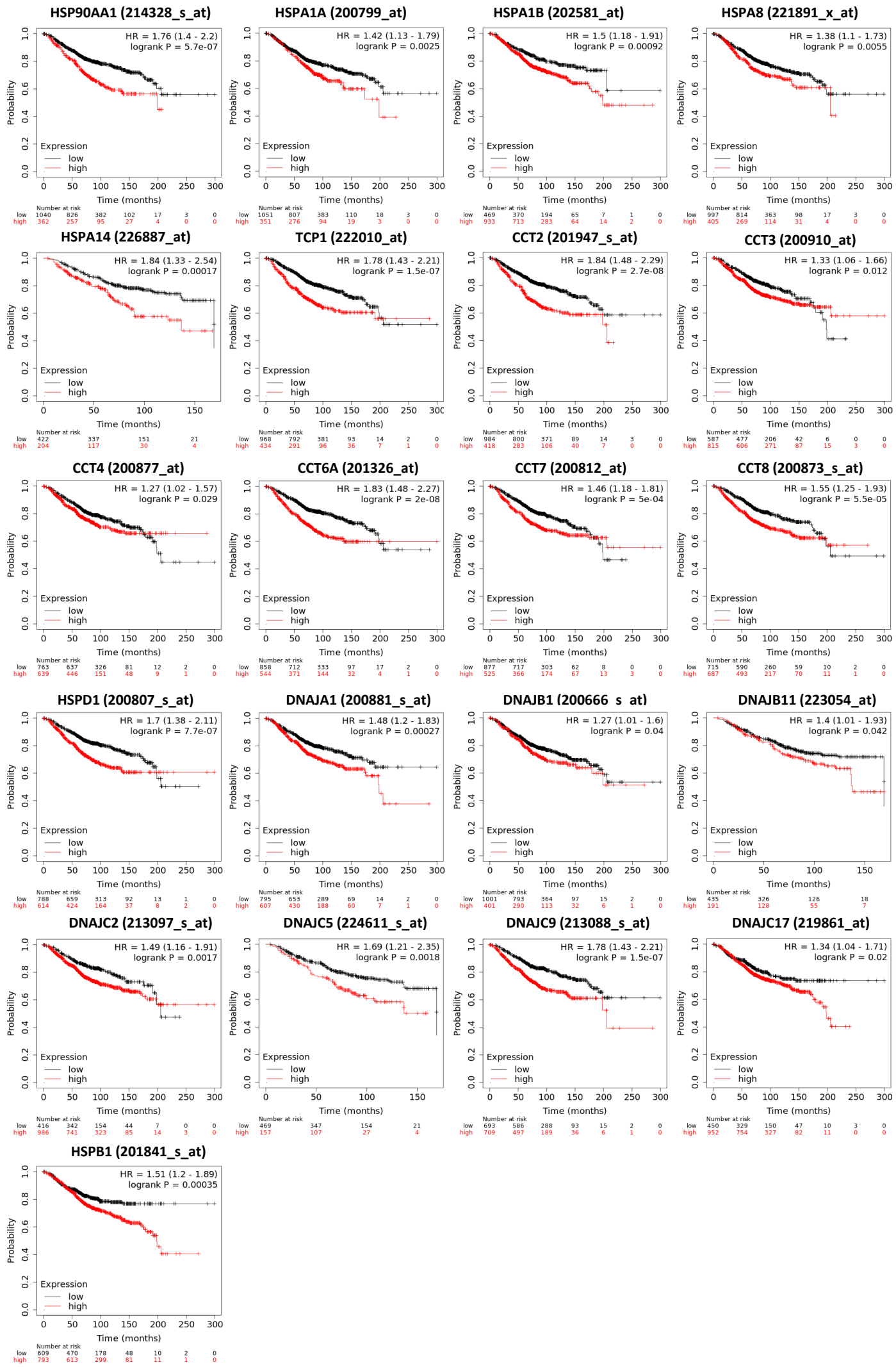

Figure S2

C

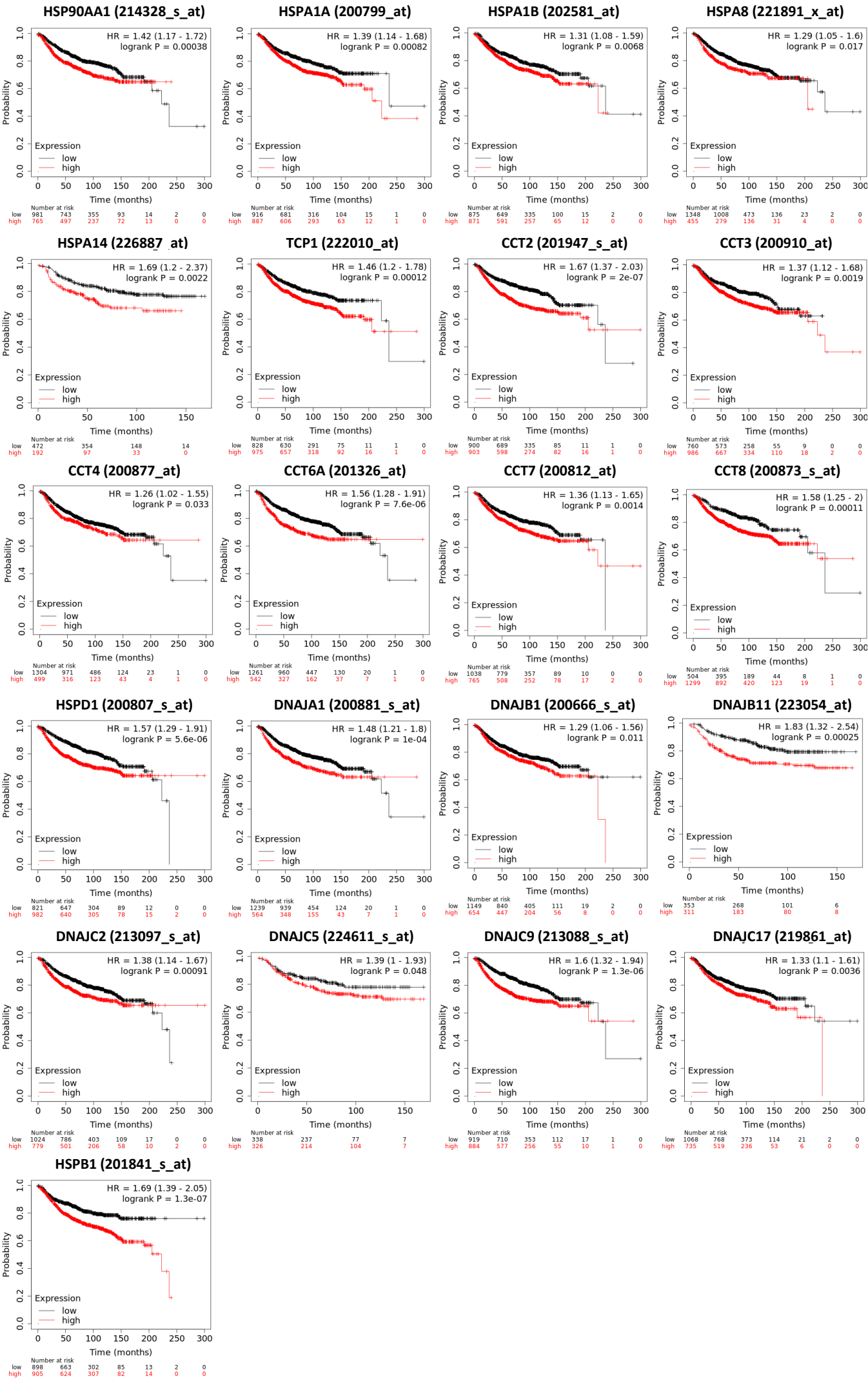

Figure S2

D

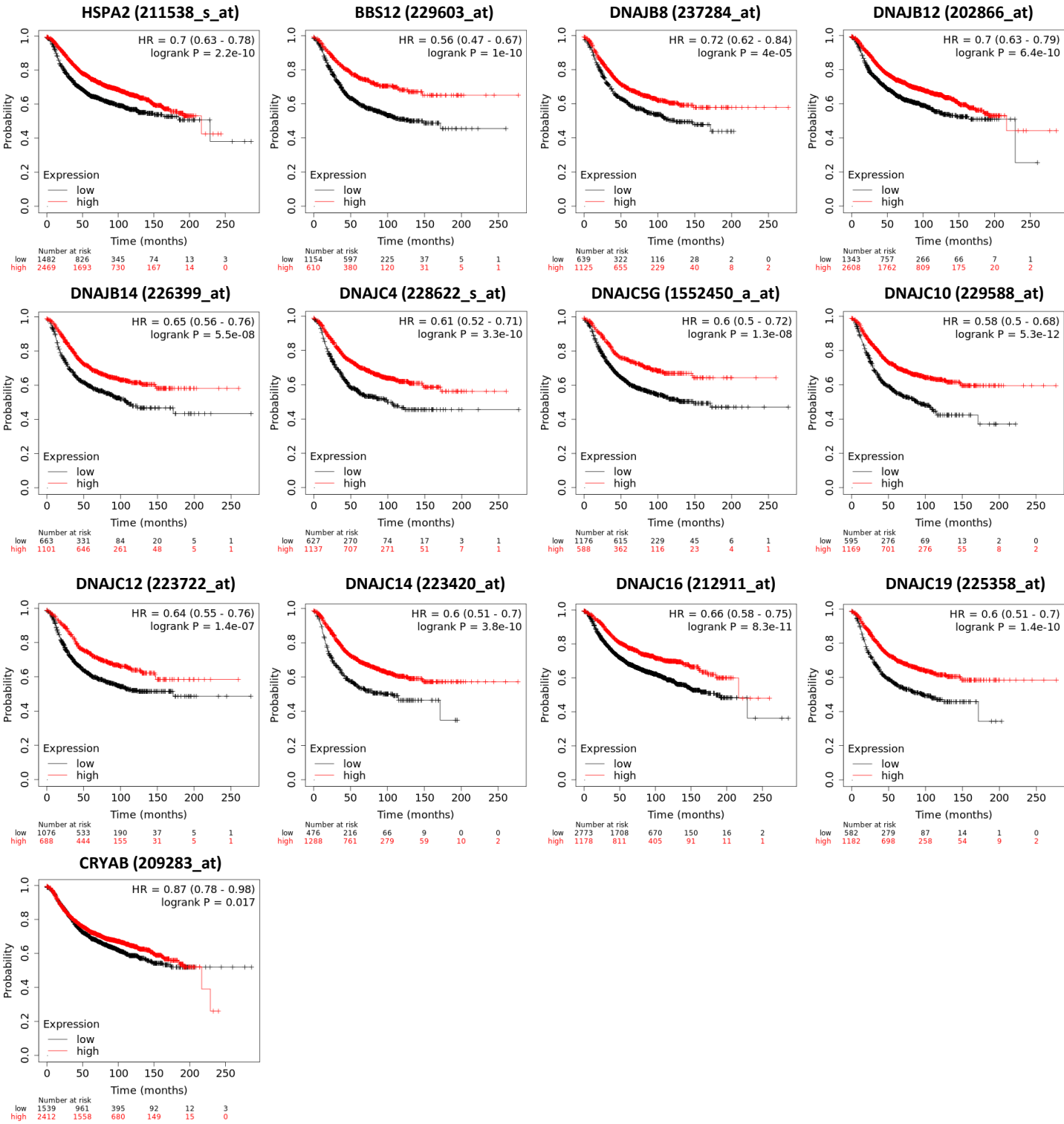

Figure S2

E

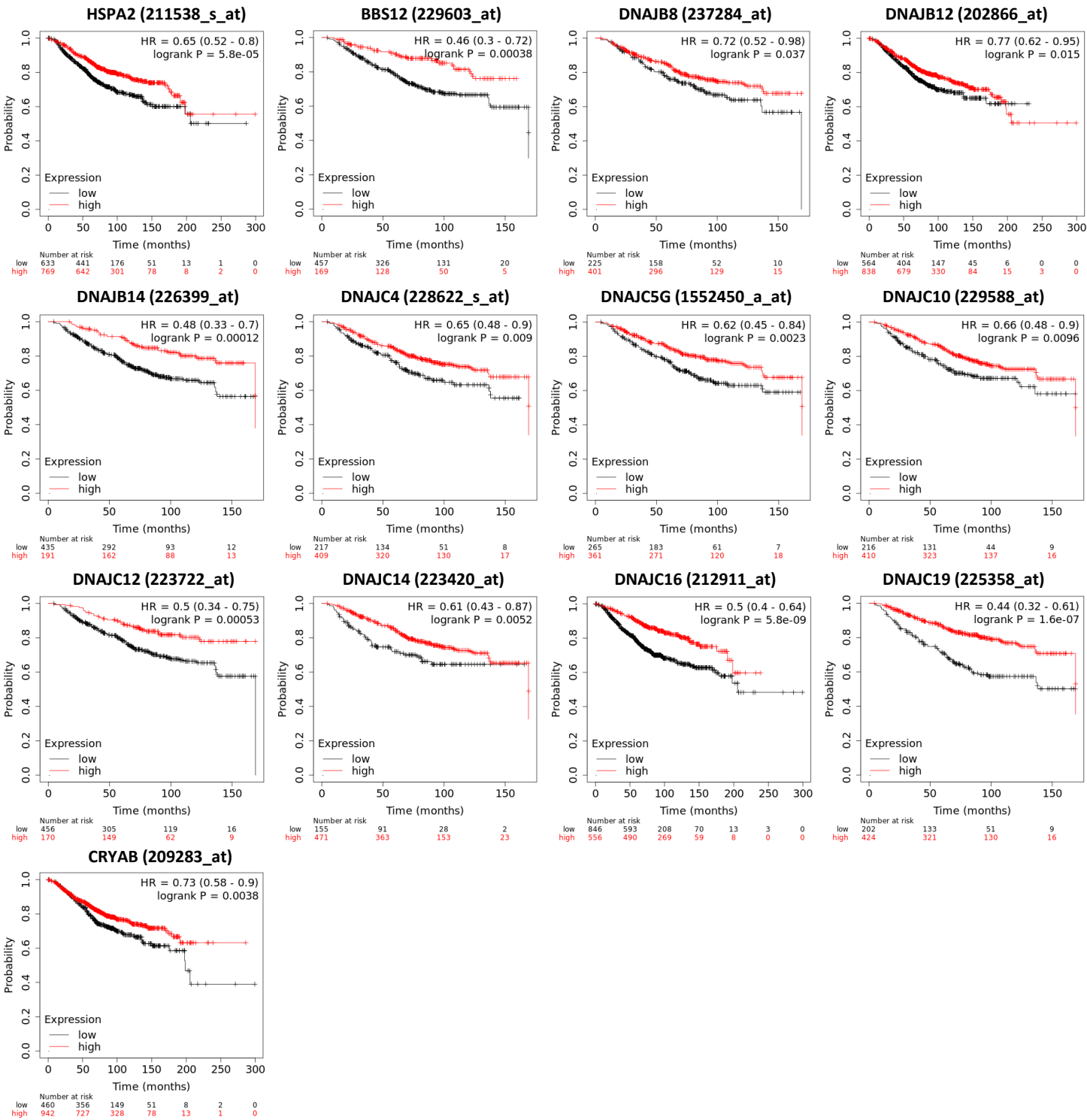

Figure S2

F

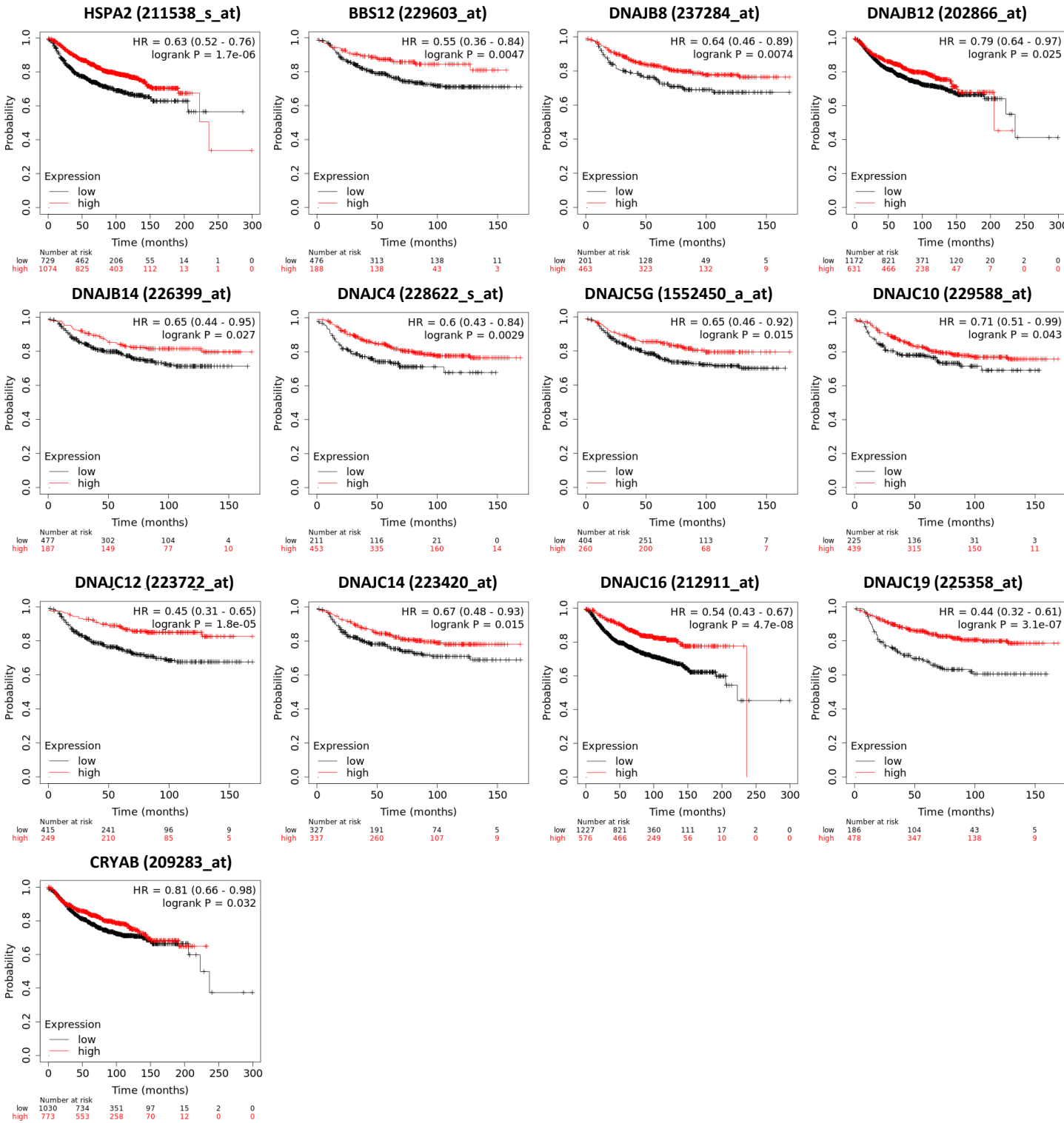

Supplement: Supplementary file 1 [file biology-10-00247-s001.zip › Supplementary/Figure S2.pdf]

Figure S3

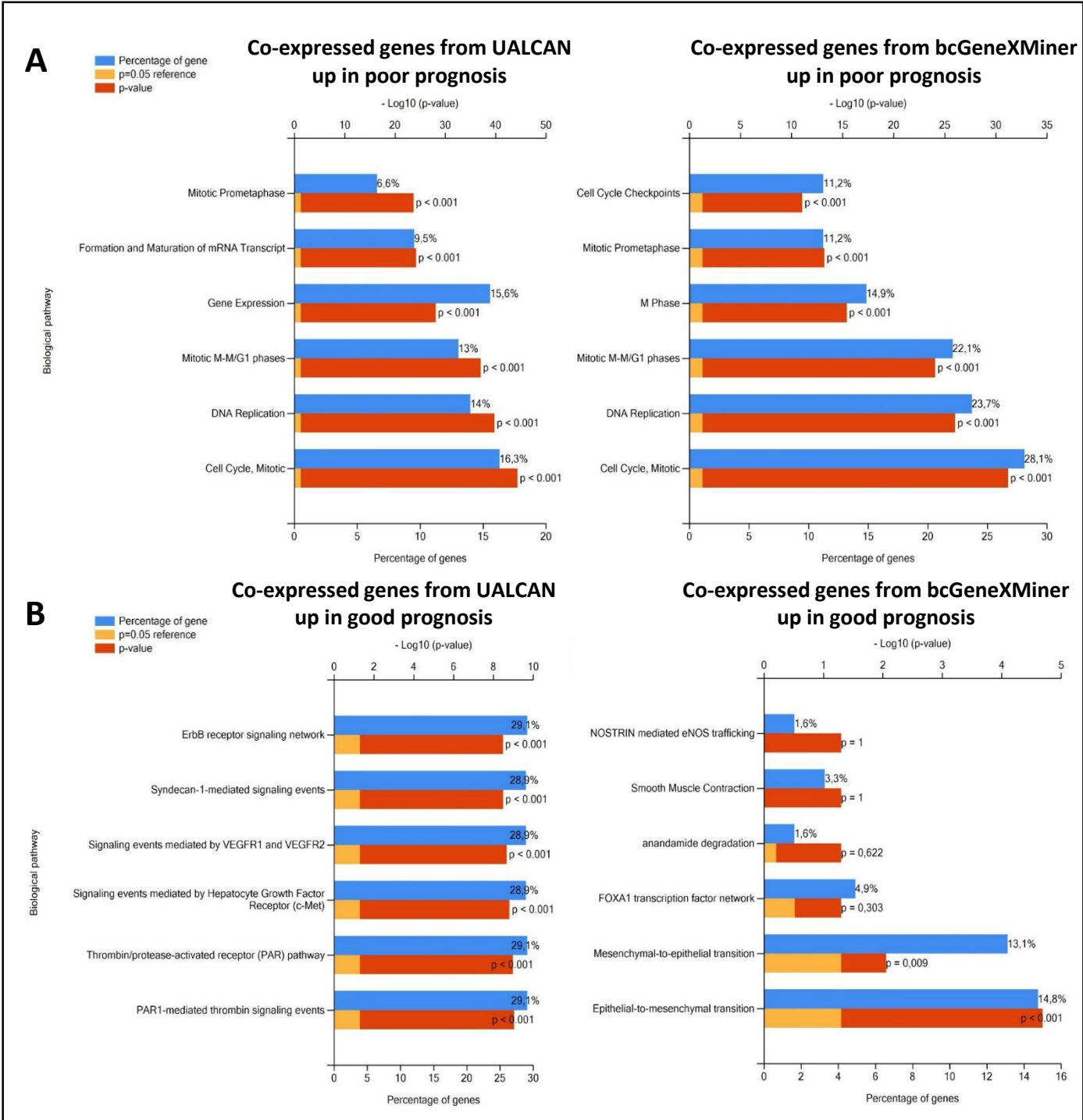

Supplement: Supplementary file 1 [file biology-10-00247-s001.zip › Supplementary/Figure S3.pdf]

Figure S4

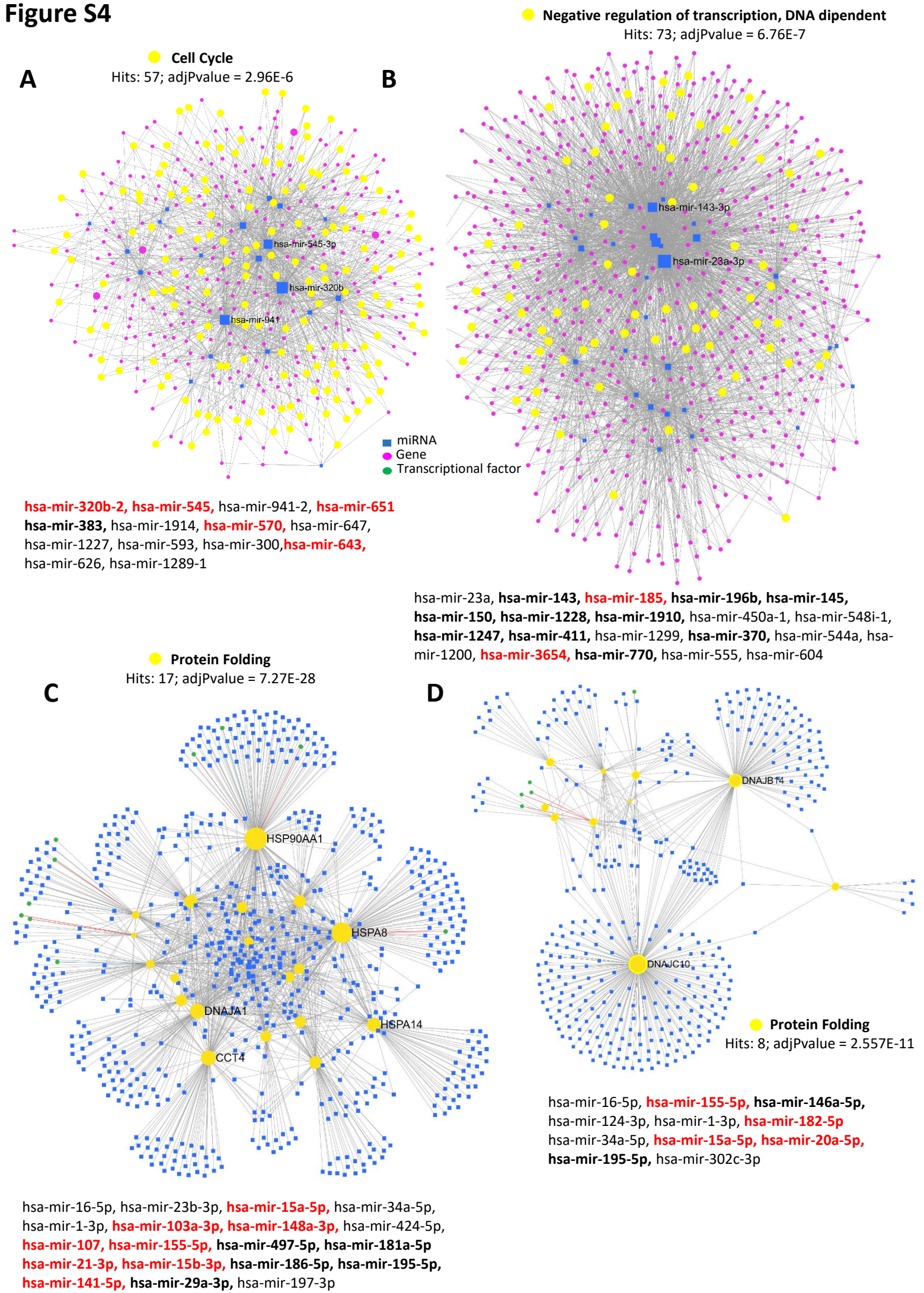

Supplement: Supplementary file 1 [file biology-10-00247-s001.zip › Supplementary/Figure S4.pdf]
