# Supplementary material for: Prognostic and Functional Significant of Heat Shock Proteins (HSPs) in Breast Cancer Unveiled by Multi-Omics Approaches
Source: Biology (Basel). 2021 Mar 22;10(3):247. doi: 10.3390/biology10030247 (PMC8004706; doi:10.3390/biology10030247)
Supplement: Supplementary file 1 [file biology-10-00247-s001.zip › Supplementary/Table S3 rev.doc]

| **Abbreviated**  **Name** | **Uniprot**  **AC** | **Mowse**  **Score** | **Number of mass**  **values matched** | **Number of mass**  **values searched** | **% Masses**  **Matched** | **% Seq.**  **Coverage** | **pI**  **exp.** | **MW (Da)**  **exp.** |
| --- | --- | --- | --- | --- | --- | --- | --- | --- |
| CH10 | P61604 | 101 | 6 | 10 | 60.00 | 40 | 9.18 | 11244 |
| CH10 a | P61604 | 115 | 8 | 20 | 40.00 | 65 | 8.30 | 11227 |
| CH60 a | P10809 | 71 | 7 | 18 | 38.89 | 16 | 5.04 | 61272 |
| CH60 b | P10809 | 206 | 27 | 47 | 57.45 | 46 | 5.09 | 61742 |
| CH60 c | P10809 | 74 | 12 | 37 | 32.43 | 21 | 5.14 | 61039 |
| CH60d | P10809 | 75 | 18 | 92 | 19.57 | 34 | 5.17 | 61507 |
| ENPL | P14625 | 74 | 12 | 15 | 80.00 | 17 | 4.73 | 90122 |
| GRP75 | P38646 | 278 | 28 | 38 | 73.68 | 43 | 5.30 | 72487 |
| GRP78 | P11021 | 75 | 8 | 24 | 33.33 | 15 | 4.90 | 75023 |
| HS71A | P08107 | 160 | 15 | 21 | 71.43 | 30 | 5.35 | 69240 |
| HSP74 | P34932 | 101 | 16 | 38 | 42.11 | 24 | 5.01 | 102944 |
| HSP74 a | P34932 | 92 | 16 | 42 | 38.09 | 26 | 5.23 | 102944 |
| HSP7C a | P11142 | 63 | 20 | 135 | 14.81 | 34 | 5.12 | 70845 |
| HSP7C b | P11142 | 68 | 14 | 60 | 23.33 | 28 | 5.16 | 70575 |
| HSP7C c | P11142 | 57 | 11 | 50 | 22.00 | 21 | 5.23 | 70306 |
| HSP7C d | P11142 | 57 | 5 | 16 | 31.25 | 11 | 5.38 | 72765 |
| HS90A | P07900 | 61 | 17 | 81 | 20.99 | 19 | 4.87 | 80980 |
| HS90B | P08238 | 68 | 11 | 32 | 34.38 | 14 | 4.94 | 80057 |
| HSPB1a | P04792 | 79 | 5 | 5 | 100.00 | 35 | 5.46 | 26008 |
| HSPB1b | P04792 | 152 | 8 | 8 | 100.00 | 40 | 5.64 | 25620 |
| HSPB1c | P04792 | 71 | 6 | 12 | 50.00 | 32 | 5.88 | 25620 |
| HSPB1d | P04792 | 125 | 12 | 32 | 37.50 | 52 | 5.99 | 26165 |
| HSPB1e | P04792 | 78 | 5 | 8 | 62.50 | 23 | 5.92 | 24713 |
| HSPB1f | P04792 | 62 | 9 | 81 | 11.11 | 33 | 5.00 | 26086 |
| HSPB1g | P04792 | 60 | 4 | 8 | 50.00 | 15 | 5.23 | 25620 |
| HYOU1 | Q9Y4L1 | 84 | 12 | 19 | 63.16 | 20 | 5.03 | 170331 |
| TCPA | P17987 | 59 | 6 | 11 | 54.55 | 13 | 5.80 | 60343 |
| TCPB | P78371 | 67 | 7 | 14 | 50.00 | 13 | 6.36 | 54275 |
| TCPE | P48643 | 85 | 9 | 38 | 23.68 | 23 | 5.35 | 60574 |
| TCPG | P49368 | 103 | 20 | 70 | 28.57 | 35 | 6.46 | 63902 |
| TCPZ | P40227 | 161 | 19 | 38 | 50.00 | 43 | 6.73 | 55427 |
| TCTP | P13693 | 97 | 11 | 26 | 42.31 | 40 | 4.75 | 21529 |

**Table S3**: List of the HSP proteins identified in the proteomics maps of breast cancer tissues by Maldi-Tof. For each protein the Abbreviated Name, the Uniprot AC, the Mowse Score, the Number of mass values matched, the Number of mass values searched, the % Masses Matched, the % Sequence Coverage, and the experimental MW (Da) and the pI are indicated.
